# The role and therapeutic advances of neutrophils in acute myocardial infarction: from traditional chinese medicine modulation to modern therapeutic strategies

**DOI:** 10.1186/s13020-025-01261-4

**Published:** 2025-12-05

**Authors:** Yisha Wan, Junping Zhang, Yingxi Yang

**Affiliations:** 1 National Institute of Traditional Chinese Medicine Constitution and Preventive Medicine , Beijing University of Chinese Medicine, Beijing, 100029 China; 2 School of Traditional Chinese Medicine, Beijing University of Chinese Medicine, Beijing, 100105 China; 3 Department of Geriatric Medicine, First Teaching Hospital of Tianjin University of Traditional Chinese Medicine, 314 An Shan Xi Road, Nan Kai District, Tianjin, 300193 China; 4 Tianjin University of Traditional Chinese Medicine, Tianjin, 301617 China; 5 Tianjin Key Laboratory of Modern Chinese Medicine Theory of Innovation and Application, Tianjin, 301617 China

**Keywords:** Acute myocardial infarction, Neutrophils, Mechanism of action, Traditional Chinese medicine

## Abstract

Acute Myocardial Infarction (AMI) is one of the leading causes of global mortality, posing a severe threat to human health. Currently, the clinical management of AMI remains a major challenge, and its pathological mechanisms lack systematic elucidation. Neutrophils, as key effector cells in the pathogenesis of AMI, have emerged as a highly promising therapeutic target. This review comprehensively summarizes the origin, subtypes, stage-specific roles, and mechanistic contributions of neutrophils in AMI. Furthermore, it explores how active components of traditional Chinese medicine (TCM) modulate neutrophil function through multi-target interventions, exerting therapeutic effects in anti-inflammation, anti-fibrosis, and pro-angiogenesis. Recent advances highlight significant progress in neutrophil-targeted therapies, including novel drug development, nanoparticle-based delivery systems, and multi-omics biomarker discovery, offering new avenues for precision medicine in AMI. Although translational challenges persist, enhanced collaboration between basic research and clinical applications may unlock innovative treatment strategies for AMI patients.

**Graphical Abstract:**

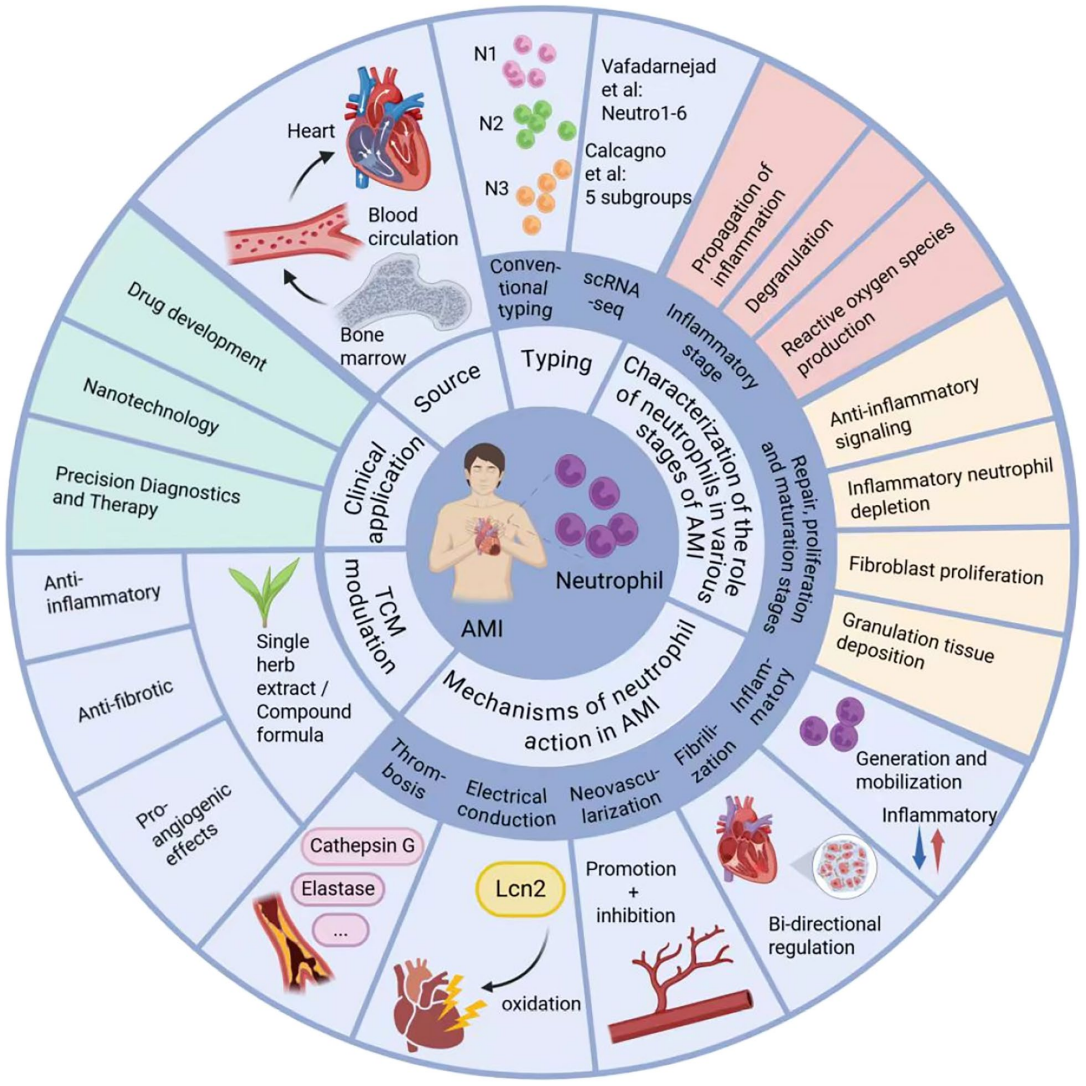

**Supplementary Information:**

The online version contains supplementary material available at 10.1186/s13020-025-01261-4.

## Introduction

Acute myocardial infarction (AMI), a life-threatening manifestation of coronary artery disease, remains the leading global cause of mortality and a major contributor to sudden cardiac death [1]. Pathologically, AMI is classified into ST-segment elevation myocardial infarction (STEMI) and non-ST-segment elevation myocardial infarction (NSTEMI). With an incidence of approximately one case every 40 s in the United States alone [2], AMI constitutes both a critical public health challenge and the most common reason for emergency department visits worldwide due to acute coronary syndromes.

The immune response plays a pivotal role in AMI pathophysiology. Neutrophils, the most abundant polymorphonuclear leukocytes in circulation, are the first immune cells to infiltrate the infarct zone [3]. These cells exert dual functions in AMI progression, participating in both inflammatory regulation and cardiac remodeling processes. Recent advances in neutrophil biology have revealed distinct subpopulations with stage-specific roles, offering new therapeutic targets for AMI management. Notably, bioactive compounds derived from traditional Chinese medicine (TCM) have demonstrated promising neutrophil-modulating effects in preclinical AMI models

Although significant progress has been made in recent years regarding the role of neutrophils in the pathological process of AMI, a comprehensive discussion that systematically elucidates their dynamic changes in the infarct zone, phenotypic transitions, and complex relationship with myocardial repair remains relatively scarce. Particularly lacking are in-depth reviews that explore strategies for synergistically regulating neutrophil function through TCM interventions from a multi-target and holistic regulatory perspective. This review aims to fill this research gap by systematically summarizing the origin, classification, stage-specific roles, and molecular mechanisms of neutrophils in AMI, with a focus on recent advances in the multi-component, multi-pathway synergistic regulation of neutrophil function by TCM. The findings are expected to provide a theoretical basis for understanding the regulation of the immune microenvironment in AMI and for developing novel therapeutic strategies.

This review systematically summarizes the role of neutrophils in AMI by elaborating on three key aspects: their origin and classification, stage-specific functional characteristics, and underlying molecular mechanisms. Furthermore, it synthesizes recent research advances concerning the regulatory effects of traditional Chinese medicine on neutrophils in the context of AMI. The aim is to provide insightful perspectives to guide future investigations and facilitate clinical translation.

## Source and typing of neutrophils in the heart

### Source

Under complex transcriptional regulation, neutrophils and other granulocytes are replenished in the bone marrow (BM) through granulopoiesis, in which hematopoietic progenitors differentiate along the neutrophil, eosinophil, or basophil lineage and develop into fully mature granulocytes [4]. Terminally differentiated neutrophils are preserved in the BM until they enter the circulation following signals from the local stromal microenvironment and are recruited to sites of injury or infection. Recent studies utilizing flow cytometry and single-cell RNA sequencing (scRNA-seq) have identified a distinct neutrophil subpopulation expressing SiglecF (SigF), a classical eosinophil surface marker. These neutrophils can spontaneously differentiate into SigF^HI^ and SigF^LO^ subsets, exhibiting dual differentiation potential, and are present in both acute ischemic injury and chronic non-ischemic injury of the heart. Emerging evidence reveals that this neutrophil heterogeneity originates in the BM, where a small subset of neutrophils already displays SigF-associated gene signatures. However, their full SigF phenotype—including surface SigF expression—is ultimately established within the local cardiac microenvironment. Infiltration of ischemic cardiac tissue is required to develop the full SigF‐associated transcriptional fingerprint and acquire surface protein expression of SigF [5].

### Typing

Following AMI, neutrophils undergo polarization and can be classified into three distinct subpopulations (N1, N2, and N3) based on their expression profiles of proinflammatory markers (C–C motif chemokine ligand 3 [Ccl3], Ccl5, and interleukin-1β [IL-1β]) and anti-inflammatory markers (arginase-1 [Arg1], CD206, and IL-10). The N1 subtype, the predominant neutrophil phenotype in the post-AMI left ventricle (comprising ~ 80% of infiltrating neutrophils), exhibits marked upregulation of chemokine genes (Ccl2, Ccl7, Ccl9, and Ccl12). This chemokine signature drives extensive immune cell extravasation into the infarct zone [6]. Notably, N1 neutrophils express matrix metalloproteinase-12 (MMP-12) and MMP-25, which exert proteolytic activity on extracellular matrix components, directly contributing to ventricular wall thinning. In contrast, the N2 subpopulation demonstrates a time-dependent expansion during AMI progression. These cells significantly upregulate interferon-stimulated genes (ISGs) and the granule protein-encoding gene Mmp8, which are critical for neutrophil-mediated host defense. Importantly, N2 neutrophils exhibit cardioprotective properties by attenuating ventricular wall thinning and mitigating adverse infarct remodeling. The N3 subset, characterized by the oldest transcriptional profile, displays enrichment in apoptosis-related pathways (e.g., Superoxide dismutase 2 [Sod2] and macrophage migration inhibitory factor [Mif]). This population facilitates the transition from the inflammatory phase to the reparative phase of myocardial infarction, effectively constraining post-infarction inflammatory responses [7].

In recent years, recognizing the limited relevance of the above classification methods to neutrophils in cardiac inflammation, researchers have adopted a new research method, scRNA-seq, to classify neutrophils, which allows for a more comprehensive description of neutrophil subpopulations and their role in the dynamics of the ischemic heart. Neutrophil classification is typically defined by the expression of established surface and intracellular markers, including S100 calcium-binding proteins (S100 Calcium Binding Protein A8 [S100A8] and S100A9), cytokine receptors (Colony Stimulating Factor 3 Receptor [CSF3R] and Interleukin 1 Receptor Type 2 [IL1R2]), chemokine receptors (C-X-C Motif Chemokine Receptor 2 [CXCR2]), proteolytic enzymes (MMP9), and hematopoietic growth factors (Colony Stimulating Factor 1 [CSF1]).Vafadarnejad et al. [8] used single-cell transcriptomics combined with cell surface epitope detection by sequencing to study the diversity of neutrophils in the blood and heart after AMI in mice. Ultimately, the researchers classified AMI-onset cardiac neutrophils into six transcriptionally distinct and time-dependent cell populations, namely Neutro1 (Tnf, Icam1, Il23a, Gpr84), Neutro2 (Slpi, Ifitm1, Wfdc17, Asprv1), Neutro3 (Rps19, Ltc4s, Nr4a2), Neutro4 (Cxcl3, Lcn2, Osm, Cd177, Ccl6, Sell, Fpr1), Neutro5 (Isg15, Rsad2, Ifit1), and Neutro6 (Psap, Slc26a11, Gdf15). Calcagno et al. [5] established a mouse model of permanently ligated left anterior descending coronary artery for single-cell RNA sequencing and analysis. With the help of various markers, they classified neutrophils into five subpopulations: high expression of Retnlg (Retnlg^HI^), interferon-stimulated genes (ISG^HI^), genes regulated by nuclear factor kappa-light-chain-enhancer of activated B cells (NFκB) (NFκB^HI^), genes regulated by hypoxia-inducible factor 1 (HIF-1^HI^), and the classically eosinophilic surface marker SiglecF (SigF^HI^). SigF is a classical marker used to define eosinophils. In a study on classical markers, Vafadarnejad et al. identified SigF^HI^, a SigF-expressing neutrophil subset, as one of the predominant neutrophil subpopulations in the cardiac tissue. Their research demonstrated that SigF^HI^ neutrophils exhibit prolonged survival within infarcted areas, characterized by upregulated oxidative phosphorylation-related gene expression and enhanced phagocytic capacity with robust reactive oxygen species (ROS) production [5, 8].

## Characterization of the role of neutrophils in various stages of AMI

**Fig.1:**
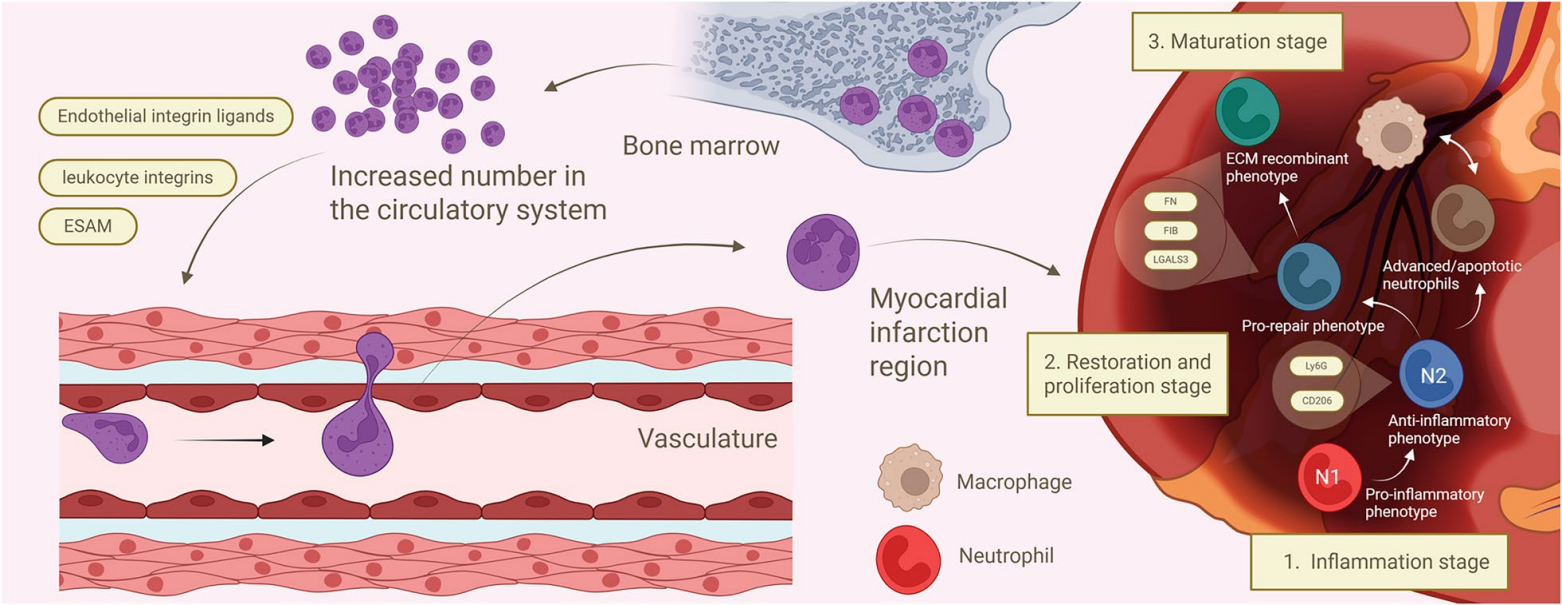
Photo of the mechanism of neutrophils in the course of AMI

As illustrated in Fig. 1, neutrophils exhibit spatiotemporal dynamics during AMI progression. In the early response phase, BM-derived neutrophils are rapidly mobilized into circulation and undergo integrin-dependent extravasation—a coordinated process involving endothelial crawling, basement membrane transmigration, and microvascular wall penetration. Chemotactic signals guide their precise homing to the infarct zone, where they exert pro-inflammatory effects (N1 phenotype) via release of matrix metalloproteinases (MMPs) and ROS. During disease evolution, neutrophils undergo phenotypic reprogramming: a subset differentiates into pro-reparative subpopulations (N2 phenotype) with extracellular matrix remodeling capacity, while others facilitate inflammation resolution via apoptotic pathways, collectively driving tissue repair.

### Inflammatory stage

Prior to AMI, cardiac neutrophils exist in a quiescent state characterized by low expression of both pro- and anti-inflammatory markers, primarily functioning in tissue surveillance [9]. Following AMI onset, neutrophils rapidly mobilize from the BM into circulation as early responders to cardiac injury. The recruitment process involves coordinated interactions between leukocyte integrins, endothelial adhesion molecules, and integrin ligands, enabling neutrophils to extravasate through the microvasculature [10]. Through exocytosis-mediated adhesion and crawling behaviors, neutrophils traverse endothelial junctions and migrate across basement membrane regions with low matrix protein expression. Upon tissue entry, chemotactic signals direct neutrophils to the infarct zone, where they execute diverse functions including inflammatory amplification, degranulation, and ROS generation [11]. In the infarct core, neutrophils primarily adopt a pro-inflammatory (N1-type) phenotype, where infiltrating cells overproduce ROS via synergistic activation of NADPH oxidase 2 (NOX2) and myeloperoxidase (MPO). These ROS not only initiate a positive feedback loop by activating redox-sensitive signaling pathways such as NF-κB, leading to the upregulation of inflammatory factors including TNF and IL-1β, but also serve as a key driver of Neutrophil Extracellular Trap-associated cell death (NETosis). Specifically, ROS promote chromatin decondensation, while MPO—released during degranulation and activated by H₂O₂—cooperates with neutrophil elastase that translocates to the nucleus to induce nuclear membrane disruption and chromatin decompaction, ultimately resulting in the release of stable neutrophil extracellular traps (NETs). Throughout this process, proteases released via degranulation and ROS collectively establish a self-amplifying inflammatory cycle [12–14]. Recruited neutrophils thus exert their functions—including phagocytosis, degranulation, ROS production, and NETosis—through these interconnected mechanisms, which serve to clear necrotic tissue at the site of myocardial injury [15]. However, excessive activation of these processes can lead to unintended tissue destruction [16]. Neutrophil infiltration predominantly localizes to the infarct border zone, with reperfusion accelerating both the rate and magnitude of recruitment. The infarct border zone, as the transitional area between viable myocardium and necrotic tissue, exhibits a dual functionality of neutrophils: On one hand, the proteolytic enzymes released during neutrophil extravasation clear necrotic cells and matrix debris, driving the transition from acute inflammation to the repair phase [17]. On the other hand, their phenotypic polarization from pro-inflammatory (N1) to anti-inflammatory (N2) further accelerates this reparative process [9]. The inflammatory phase ultimately culminates in extracellular matrix remodeling and apoptotic cell clearance [18].

### Repair, proliferation and maturation stages

The inflammatory phase is succeeded by sequential repair, proliferation, and maturation stages. The repair/proliferation stage, typically occurring 7–10 days post-MI, features anti-inflammatory signaling, neutrophil clearance, fibroblast activation, and granulation tissue formation [19]. This transition is mediated by dynamic changes in the cardiac microenvironment [10]. During this phase, late-stage apoptotic neutrophils facilitate inflammation resolution through multiple mechanisms [7]. Notably, a neutrophil subpopulation undergoes phenotypic switching to a pro-reparative state, characterized by elevated expression of fibronectin, galectin-3, and fibrinogen, which orchestrates extracellular matrix (ECM) remodeling to support scar formation [9]. These ECM components (including fibronectin, vitronectin, and fibrinogen) establish a provisional matrix that guides cellular migration and proliferation during wound healing [10]. The reparative neutrophil subsets (N2/N3) uniformly express lymphocyte antigen 6 complex (Ly6G) and CD206 (mannose receptor), which mediate phagocytic clearance of apoptotic cells and debris to promote myocardial healing [20].

Simultaneously, apoptotic neutrophils initiate the wound-healing phase of cardiac remodeling by promoting macrophage polarization toward an anti-inflammatory M2 phenotype via direct cellular crosstalk [21]. Pro-inflammatory neutrophils contribute to this process through targeted degranulation, releasing matrix metalloproteinases that selectively degrade the peri-necrotic extracellular matrix—an essential prerequisite for debris clearance and subsequent tissue repair [22]. These neutrophils further orchestrate post-MI healing through paracrine signaling that reprograms macrophages toward a pro-reparative phenotype, thereby indirectly modulating left ventricular remodeling [21]. This coordinated healing response culminates in robust scar formation that prevents mechanical complications (including myocardial rupture) while preserving ventricular function [16].

The maturation phase progresses over subsequent months, during which neutrophils adopt a pro-reparative phenotype characterized by enhanced expression of ECM-modulating factors (fibronectin, galectin-3) and active participation in collagen cross-linking-mediated ECM reorganization [16]. Inadequate ECM remodeling during this phase may precipitate pathological myocardial stiffening, impaired diastolic function, and ultimately heart failure development [10]. This neutrophil-mediated remodeling process therefore represents a critical determinant of functional cardiac recovery (Fig. 1).

## Mechanisms of neutrophil action in AMI

### Neutrophils and the cardiac inflammatory response

**Fig.2:**
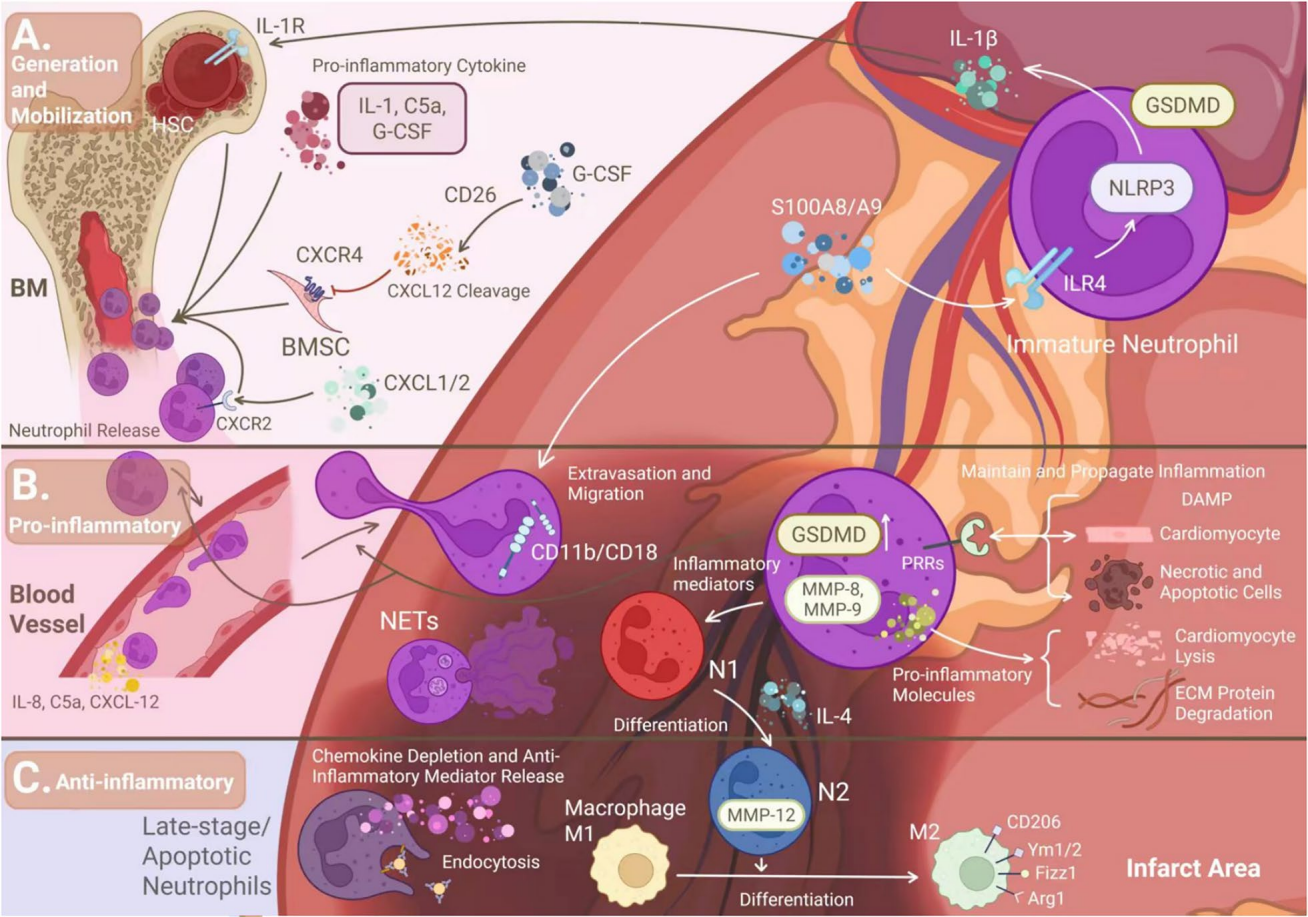
Neutrophil-cardiac inflammation interactions. **A** Neutrophil production and mobilization during inflammatory responses. **B** Pro-inflammatory functions of neutrophils in the infarct zone. **C** Anti-inflammatory roles of neutrophils in the infarct zone

Neutrophils represent the most abundant leukocyte population and serve as first responders in initiating both innate and adaptive immune responses during inflammatory challenges. As illustrated in Fig. 2, neutrophil dynamics post-AMI progress through three distinct phases: bone marrow generation and mobilization (Fig. 2A), pro-inflammatory effector functions in the infarct zone (Fig. 2B), and anti-inflammatory reparative actions (Fig. 2C). The robust infiltration of leukocytes into ischemic myocardium constitutes a defining characteristic of post-AMI inflammation [6], with neutrophils emerging as the predominant immune cell type within the infarct zone [23]. Following AMI, neutrophils undergo a well-orchestrated temporal response: initial recruitment to the infarcted region promotes inflammatory activation and initiates wound repair processes. During this early phase, pro-inflammatory neutrophils facilitate ECM degradation through protease secretion, enabling clearance of necrotic cardiomyocytes. Subsequently, these cells undergo phenotypic modulation, acquiring anti-inflammatory and pro-reparative functions that drive the transition from active inflammation to resolution and scar maturation.

While controlled neutrophilic inflammation is essential for effective debridement of necrotic tissue and initiation of repair mechanisms, excessive or sustained inflammatory activation—particularly in cases of extensive infarction—predisposes to progressive ventricular dilation, persistent immune cell infiltration [10], and subsequent heart failure development. Such dysregulated inflammatory responses may precipitate maladaptive left ventricular remodeling, increasing susceptibility to ventricular arrhythmias (VA) and contributing significantly to AMI-related morbidity and mortality. These pathophysiological insights highlight the critical need to elucidate neutrophil-mediated inflammatory regulation in AMI, as understanding these mechanisms may yield novel therapeutic strategies. This review systematically examines the dynamic interplay between neutrophils and cardiac inflammation throughout distinct phases of AMI progression.

#### Neutrophil production and mobilization in relation to inflammation

Neutrophil production and mobilization are tightly regulated by inflammatory mediators. AMI triggers the release of proinflammatory cytokines—including interleukin (IL)−1, complement component 5a (C5a), and Granulocyte colony-stimulating factor (G-CSF)—that promote neutrophil egress from BM reservoirs. The C-X-C motif chemokine ligand 12 (CXCL12)-CXCR4 axis serves as a critical retention system, where CXCL12 binding to CXCR4 on BM stromal cells maintains neutrophil quiescence [11]. G-CSF-mediated cleavage of CXCR4 through CD26 peptidase activity disrupts this retention signal, facilitating neutrophil release into circulation [24]. Experimental evidence from permanent left anterior descending artery ligation models demonstrates that myocardial infarction rapidly recruits neutrophils to ischemic myocardium. These activated neutrophils secrete S100A8/A9 alarmins that engage toll-like receptor 4 (TLR4) and activate the nod-like receptor family pyrin domain-containing 3 inflammasome (NLRP3) inflammasome in resident neutrophils, triggering IL-1β production [23]. The cytokine subsequently binds IL-1 receptors on BM hematopoietic stem cells (HSC), autonomously enhancing granulopoiesis. This S100A8/A9-TLR4-IL-1β axis constitutes a positive feedback loop that amplifies neutrophil mobilization post-AMI [25]. Complementing these mechanisms, the CXCL1/2-CXCR2 chemokine axis further drives neutrophil recruitment [26]. CXCL1 and CXCL2 function as potent neutrophil chemoattractants that activate CXCR2 signaling, ultimately promoting BM neutrophil release [27].

#### Neutrophils exert a pro-inflammatory effect at the site of infarction

Neutrophils exert pro-inflammatory effects in the infarcted myocardium through multiple coordinated mechanisms. The integrin CD11b/CD18 (Mac-1/CR3/αMβ2), expressed on neutrophils and other leukocytes, mediates endothelial adhesion and transvascular migration during inflammatory responses [28]. Neutrophils amplify inflammation through interactions with damage-associated molecular patterns (DAMPs) released from necrotic cardiomyocytes post-AMI [29]. These endogenous danger signals bind pattern recognition receptors on leukocytes, triggering complement activation and cytokine production while sustaining the inflammatory cascade [30]. DAMPs induce neutrophil polarization toward a pro-inflammatory N1 phenotype characterized by elevated expression of Ccl3, Il1β, Il12a, and TNF-α [10]. This process involves gasdermin D (GSDMD)-dependent inflammasome activation, which facilitates IL-1β release and exacerbates acute inflammation [31]. Notably, neutrophil recruitment is mediated by chemotactic factors including IL-8, CXCL12, and C5a, with emerging evidence suggesting sex-specific differences in these signaling pathways [11]. Neutrophil-derived proteolytic enzymes contribute significantly to tissue injury. Granule contents such as matrix metalloproteinases (MMP-8/9) modulate inflammation through cytokine processing and extracellular matrix degradation [32]. Early elastase release induces IL-6 secretion via nitric oxide-dependent mechanisms, impairing myocardial contractility [33, 34]. Furthermore, NETs—chromatin networks decorated with granular proteins—perpetuate inflammation and correlate with adverse clinical outcomes including larger infarct size and worsened ventricular function [14].

#### Neutrophils exert an anti-inflammatory effect at the site of infarction

Neutrophils exhibit a dual regulatory role in inflammation, demonstrating both pro-inflammatory and anti-inflammatory functions. The resolution of inflammation, essential for restoring tissue homeostasis, is characterized by neutrophil clearance from myocardial tissue [6]. Late-stage and apoptotic neutrophils promote inflammation resolution through several mechanisms, including the release of pro-resolving mediators, downregulation of chemokine and cytokine receptors, and enhanced endocytic activity [10]. During the later phases of AMI, neutrophils undergo phenotypic switching from the pro-inflammatory N1 phenotype to the anti-inflammatory N2 phenotype following IL-4 stimulation, accompanied by increased expression of anti-inflammatory markers. Neutrophil-derived matrix metalloproteinase-12 (MMP-12) contributes to inflammation resolution by modulating inflammatory signaling pathways and facilitating cellular clearance [16]. Additionally, neutrophils regulate the inflammatory response through cytokine secretion and protease-mediated cytokine processing [10]. A critical function of neutrophils in post-AMI healing involves their ability to promote macrophage polarization toward the reparative M2 phenotype, which is indispensable for proper cardiac tissue repair [6].

### Neutrophils and cardiac fibrosis

Following AMI, the infarcted myocardium, characterized by extensive cardiomyocyte necrosis due to perfusion deficits and limited regenerative capacity, faces an imminent risk of rupture [35]. In this context, neutrophils play a pivotal and dual regulatory role as a central hub that determines the repair outcome by modulating the dynamic balance between ECM degradation and synthesis. Their function extends beyond simple promotion or inhibition, critically relying on the precise temporal regulation of ECM degradation and synthesis to dictate the final repair result. During the early phase, neutrophils efficiently clear damaged matrix by secreting various proteases and finely regulating NETs, thereby creating space for tissue remodeling and preventing the pathological accumulation of provisional matrix. In the subsequent repair phase, they activate cardiac fibroblasts through direct or indirect pathways, driving the synthesis of collagen-based protective scarring. Ultimately, the progression of cardiac fibrosis toward adaptive healing or pathological remodeling directly depends on the spatiotemporally precise coordination and dynamic interplay between these two processes.

#### NETs in early fibrosis clear damaged matrix

During the acute inflammatory phase, neutrophils play a key role in preventing excessive fibrosis by clearing damaged extracellular matrix. On one hand, neutrophil-secreted NETs are tightly regulated by Deoxyribonuclease (DNase) released from macrophages and local muscle cells; the ability of DNase to prevent sustained pathogenic fibrosis effectively inhibits myocardial fibrosis [36]. On the other hand, NETs secreted via NETosis during the early stage of AMI contribute to the degradation of adhesive proteases. Meanwhile, various proteases secreted by neutrophils serve as key tools for the clearance function: neutrophil elastase released in the early ischemic phase possesses the ability to degrade elastin [33, 34]; neutrophil-derived granule MMP-8 cleaves type I α1 and α2 chains, degrades collagen, and promotes neutrophil migration, while deficiency of MMP-8 reduces neutrophils and inhibits early collagen degradation [37]; moreover, overexpression of neutrophil-derived MMP-9 can inhibit ECM synthesis and attenuate inflammatory responses, thereby indirectly reducing myocardial fibrosis [38].

#### Matrix metalloproteinases and cytokines drive scar maturation during the repair phase

As the pathological process progresses into the intermediate and late stages, neutrophils actively drive new matrix synthesis and scar maturation, becoming a major promoter of the fibrosis process. Following AMI, neutrophils are recruited to the infarct zone to regulate cardiac fibroblasts, initiating this process: leading fibroblasts to produce an initial provisional ECM rich in fibrin or fibronectin, which subsequently shifts function to synthesize a mature collagen-based scar [39]. In the chronic phase after large-area AMI, low-quality and sustained DAMPs induce limited neutrophil infiltration and NETs secretion, promoting myocardial fibrogenesis [36]. Some neutrophils even directly participate in matrix construction, expressing fibronectin mRNA and reorganizing the initiating ECM, resulting in increased production of fibrinogen and fibronectin [9]. Subsequently, the ECM is further strengthened through enzymatic cross-linking, reinforcing the scar and preventing rupture. Fibrinogen activates fibroblasts during the wound healing process and plays an important role in post-AMI cardiac remodeling and fibrosis [40].

Neutrophils also precisely regulate this process through key cytokines. IL-1β is an inflammasome responsible for activating cardiac fibroblasts, and it creates a pro-inflammatory microenvironment in fibroblasts, thereby impairing their repair capacity [41]. Neutrophil-dependent upregulation of IL-1β alters the fibroblast phenotype, promotes provisional matrix synthesis, delays mature scar formation, and consequently leads to excessive cardiac fibrosis and increased collagen content [21, 42]. Concurrently, neutrophils can induce rapid upregulation of TGF-β1, whose activated signaling triggers excessive interstitial myocardial fibrosis and increases collagen synthesis via activation of PPAR-δ and angiotensin II [43].

Myeloperoxidase (MPO), a major neutrophil protease, has also been demonstrated to possess strong pro-fibrotic capability [41].

### Neutrophils and neovascularization

Angiogenesis constitutes a highly regulated process involving coordinated interactions between endothelial cells, smooth muscle cells, ECM components, and angiogenic factors [44]. Following AMI, this reparative process initiates in the border zone before extending toward the necrotic core. The resulting capillary network serves dual functions: facilitating leukocyte recruitment while improving oxygen delivery, nutrient supply, and metabolic waste clearance—collectively mitigating cardiomyocyte dysfunction in peri-infarct regions [45, 46]. Neutrophils emerge as key modulators of post-AMI angiogenesis through multifaceted mechanisms. MMP-9 derived from neutrophils mediates endothelial progenitor cell (CD34 + EPC) mobilization and stem cell recruitment during hypoxic conditions [47]. Furthermore, neutrophils establish a fibronectin-MMP-9 feedback loop that enables targeted wound homing and repair processes [9]. Paradoxically, early neutrophil-derived MMP-9 activity may exacerbate left ventricular dysfunction through excessive ECM degradation, thereby impairing angiogenesis during initial stages of AMI [48]. Recent investigations by Ma et al. [49] demonstrate that genetic ablation of FUN14 domain-containing 1 (FUNDC1) enhances neutrophil migration following ischemia–reperfusion (I/R) injury, consequently disrupting coronary endothelial integrity and compromising neovascularization capacity.

### Neutrophils and cardiac electrical conduction

The cardiac electrical conduction system is essential for maintaining normal heart rhythm and contractile function. Following AMI, the normally homogeneous myocardial substrate becomes electrophysiologically heterogeneous, creating pathways for electrical re-entry [50]. This arrhythmogenic substrate arises from multiple pathological alterations, including oxidative modification of ion channels and gap junctions, genetic abnormalities, and the replacement of functional myocardium with fibrotic or necrotic tissue that impairs electrical propagation [51]. These changes predispose to various life-threatening arrhythmias, which represent both a hallmark of heart failure progression and a major cause of sudden cardiac death in AMI patients. The spectrum of post-AMI arrhythmias encompasses both ventricular and supraventricular disturbances. Ventricular arrhythmias range from benign accelerated idioventricular rhythm and premature ventricular contractions to malignant ventricular tachycardia and fibrillation. Supraventricular manifestations include sinus node dysfunction (bradycardia or tachycardia), atrial fibrillation/flutter, and advanced atrioventricular block. Experimental evidence demonstrates that neutrophil depletion significantly reduces ventricular tachycardia incidence [52], implicating neutrophils in arrhythmogenesis. Mechanistically, the neutrophil-derived protein lipocalin-2 (LCN2) promotes electrical instability through oxidative modulation of ion channel function, resulting in action potential prolongation, calcium handling abnormalities, and delayed afterdepolarizations that facilitate reentrant tachyarrhythmias and fibrillation [53].

### Neutrophils and thrombosis

AMI results from the formation of occlusive thrombi within coronary arteries, leading to cardiac ischemia and subsequent infarction. The pathogenesis of AMI is driven by lipid accumulation in the arterial wall, chronic inflammation, and vascular injury. As the disease progresses, certain plaques undergo destabilization, transitioning into an unstable phenotype characterized by an intense inflammatory response. Ultimately, plaque rupture exposes the subendothelial matrix and plaque contents to circulating blood, precipitating occlusive thrombus formation. This process manifests clinically as angina, cardiomyocyte necrosis, and impaired cardiac function [54].

Research indicates that activated platelets, neutrophils, and erythrocytes play pivotal roles in thrombosis. Microscopic analysis of thrombi extracted from AMI patients by Sisakian et al. [55] revealed a structural framework composed of fibrin and densely aggregated activated platelets, surrounded by infiltrating neutrophils. Following AMI onset, platelet-derived bioactive mediators promote neutrophil adhesion to the inflamed endothelium, further exacerbating thrombogenesis. Neutrophils contribute to coagulation through multiple mechanisms, including the release of matrix metalloproteinases, platelet-activating factor, cathepsin G, and elastase. These factors modulate coagulation cascades by activating factors V, VIII, and X, enhancing platelet aggregation, degrading antithrombin III, and cleaving tissue factor (TF) pathway inhibitors.

MicroRNAs (miRNAs), short non-coding RNAs abundant in neutrophils, have been implicated in STEMI. Cardiomyocyte necrosis post-AMI may lead to miRNA leakage into the circulation [56]. Emerging evidence suggests that miRNAs regulate neutrophil migration, suppress NET formation, mitigate endothelial injury, and attenuate prothrombotic states [57]. The critical contribution of neutrophils to thrombosis also critically involves the generation of NETs. NETs are recognized as pivotal mediators in triggering coagulation through the intrinsic pathway [58]. Specifically, NET components, such as DNA, directly bind and activate factor XII (FXII) via their negatively charged surfaces, thereby initiating the intrinsic coagulation cascade. This mechanism is supported by evidence demonstrating that FXII inhibition significantly attenuates NET-induced thrombin generation. Furthermore, NETs serve as an efficient physical scaffold that concentrates coagulation factors, including fibrinogen and factor X, into localized high-density reaction centers. This promotes fibrin formation and cross-linking, thereby reinforcing thrombus stability. Thus, NETs drive thrombosis mediated by the intrinsic pathway through synergistic mechanisms: direct activation of FXII and provision of a procoagulant scaffold. Beyond direct contact activation, NETs potently initiate the intrinsic coagulation pathway through platelet-mediated amplification and the disruption of inhibitory checks. Specifically, histones on NETs activate platelets, prompting the release of polyphosphate (polyP), a potent FXII activator that launches the intrinsic cascade. Concurrently, neutrophil serine proteases (e.g., elastase) associated with NETs degrade tissue factor pathway inhibitor (TFPI), removing a major brake on coagulation initiation. Furthermore, activated platelets provide a catalytic phospholipid surface for tenase and prothrombinase complex assembly, while generated thrombin propagates clotting via a feedback loop by activating FXI. Thus, NETs drive thrombosis by synergistically activating FXII, amplifying the coagulation signal, and dismantling key regulatory mechanisms [59].

Furthermore, patients with STEMI exhibit a significantly higher burden of NETs compared to those with NSTEMI. Mechanistically, the infarct-related arteries of STEMI patients show markedly elevated levels of core NET components—double-stranded DNA (dsDNA) and MPO—indicating robust neutrophil activation and complete NETosis, leading to the formation of prothrombotic web-like structures. In contrast, NSTEMI patients demonstrate only elevated MPO without significant changes in dsDNA, suggesting partial neutrophil activation without large-scale NET formation. This discrepancy provides a pathophysiological basis for the development of complete and stable coronary thrombi mediated by NETs in STEMI. Clinically, higher NET content in thrombi from STEMI patients is closely associated with distal embolization and the no-reflow phenomenon during percutaneous coronary intervention, ultimately contributing to adverse clinical outcomes. These findings underscore NET burden as a critical biological indicator for distinguishing thrombotic characteristics and prognosis between these two types of acute myocardial infarction [60].

Post-AMI, neutrophils release NETs composed of chromatin, histones, and proteolytic enzymes. The interaction between NETs and fibrinogen exacerbates thrombosis and contributes to the "no-reflow" phenomenon, impairing coronary microvascular perfusion [61]. Furthermore, Mangold et al. [62] demonstrated that NETs are key mediators of microvascular obstruction (MVO) in STEMI patients.

## Regulatory effects of single herb extracts and compound formulations of TCM on neutrophils in AMI

**Fig. 3:**
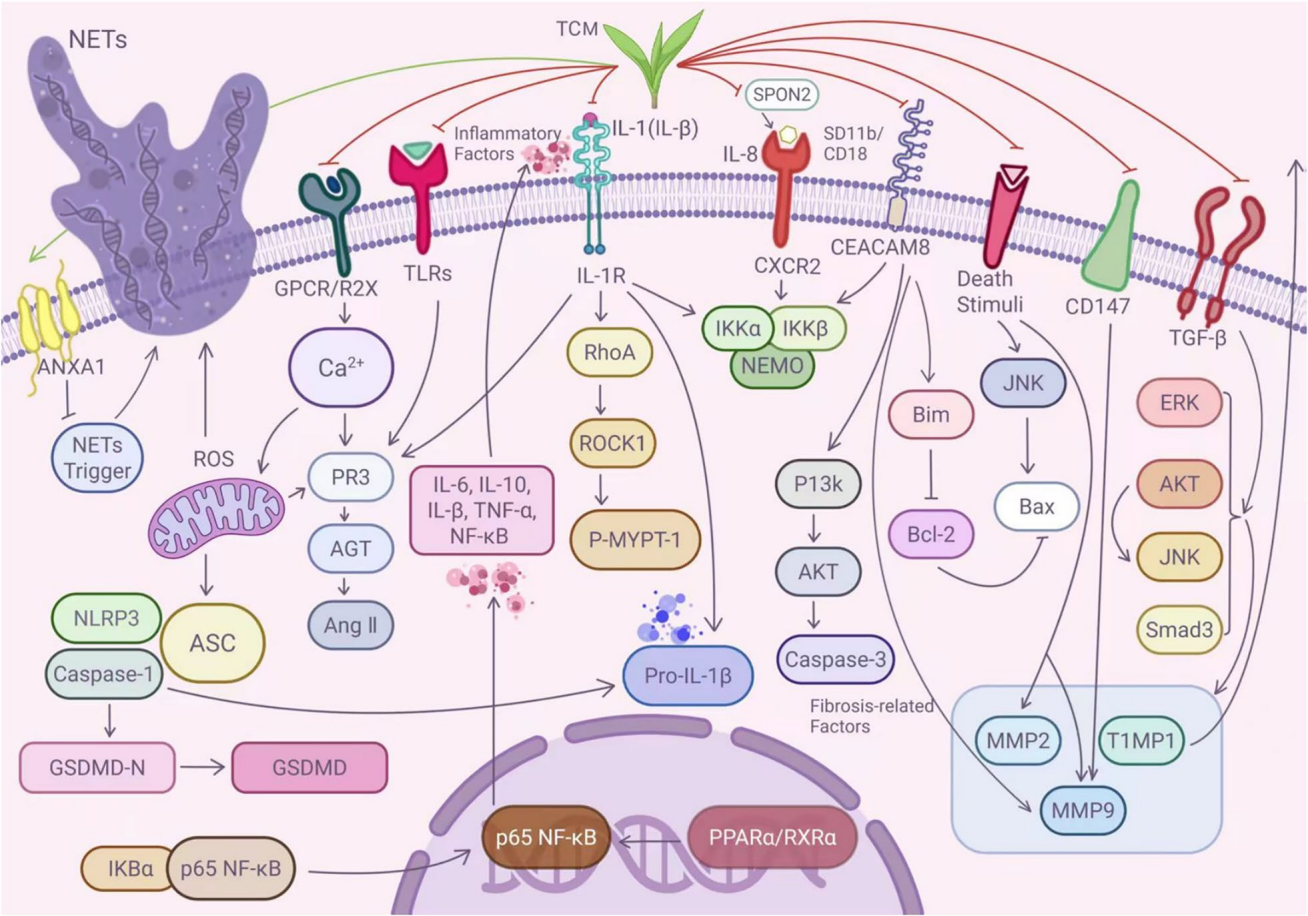
Regulatory mechanisms of single-herb extracts or compound formulations on neutrophils in AMI

Recent studies have revealed that various TCM components, particularly colchicine and curcumin (CC), exhibit regulatory effects on neutrophils in AMI, with research focusing on their anti-inflammatory and anti-fibrotic properties. With the advancement of modernization research in TCM, increasing attention has been paid to the pivotal role of neutrophils in the pathological process of AMI. This article systematically reviews recent progress in TCM intervention strategies targeting neutrophils, including both single herb extracts (Table 1) and compound formulations (Table 2), along with an illustrated mechanistic diagram (Fig. 3). The review aims to provide novel perspectives and theoretical foundations for modern research on TCM-based prevention and treatment of AMI.

**Table 1:** Therapeutic effects of single-herb extracts targeting neutrophils in AMI

Source (latin name)	Extract	Sample size	Intervention model	Control treatment	Experimental treatment	Outcomes	Mechanisms	Pathway	Refs
Anti-inflammatory									
*Astragalus membranaceus (Fisch.) Bunge*	AS-IV	–	LAD ligation	0.5% CMC (20 mL/kg/day, oral)	AS-IV (40 mg/kg/day, oral)	Inflammatory infiltration, myocardial injury, cardiomyocyte hypertrophy, myocardial fibrosis, and cardiac remodeling ↓	ROS↓, neutrophil expression↓	ROS/Caspase-1/GSDMD	[63]
*Curcuma longa L*	Curcumin	56	Isoproterenol-induced MI	Saline (s.c.)	CCNP (100/150 mg/kg/day, oral)	Cardiomyocyte necrosis, edema formation, and inflammatory cell infiltration ↓	Antioxidant response↑ (inhibition of Malondialdehyde [MDA], Total Oxidant Status [TOS], and Nitrogen Oxides [Nox] levels), pro-inflammatory cytokines (e.g., TNF-α, IL-6, IL-1α, IL-1β) serum levels↓, MMPs (MMP-2, MMP-9) expression↓	NOS/NO	[64]
*Poria cocos (Schw.) Wolf*	PCP	80	LAD ligation/reperfusion	Saline (oral)	PCP (100/200 mg/kg/day, oral)	Focal necrosis in myocardial tissue, cardiomyocyte swelling, unclear boundaries, and neutrophil infiltration ↓	IL-1β↓, IL-18↓, neutrophil adhesion↓, oxygen free radical production↓	Rho-ROCK	[65]
*Colchicum autumnale L*	Colchicine	–	LAD ligation	Cl-amidine (10 mg/kg/day)	Colchicine (0.1 mg/kg/day, i.p.)	Inflammatory response, NETs formation, and cardiac remodeling ↓	NOX2/ROS↓, Ca^2^⁺ influx↓, NETs formation↓	–	[14]
		5872	Clinical AMI	Placebo	0.5–1.0 mg/day	Risk of MACE ↓ with improved cardiovascular prognosis	C-Reactive Protein (CRP)↓, neutrophil levels↓	–	[66]
*Rabdosia rubescens (Hemsl.) H. Hararubescens*	Oridonin	–	LAD ligation	Ori/MCC950/CY-09	Saline	Neutrophil infiltration, myocardial infarction area, and myocardial fibrosis ↓	IL-1β↓, IL-18↓, inflammatory infiltration of myocardial neutrophils↓	–	[67]
*Salvia miltiorrhiza Bge*	STS	56	LAD ligation	Saline (i.p.)	STS (20.8 mg/kg/day, i.p.)	Angiogenesis ↑, cardiac function ↑, myocardial necrosis ↓, inflammatory cell infiltration ↓, scar size ↓	α-Smooth Muscle Actin (α-SMA)↓, Collagen I & III↓, inflammatory cell infiltration↓, plasma LDH↓, High Mobility Group Box 1 (HMGB1)↓, Interleukin-1β (IL-1β)↓, Tumor Necrosis Factor-α (TNF-α)↓, and protein expression of these cytokines↓	–	[68]
	Tanshinones	96	ISO-induced AMI	IMST (6.3 mg/kg)	SSPA/NSPA (1/4 g/kg, oral)	Inflammatory cell infiltration ↓, ECG ST-segment elevation ↓, myocardial infarction area ↓	Aspartate Aminotransferase (AST)↓, Lactate Dehydrogenase (LDH)↓, Creatine Kinase-MB (CK-MB)↓, Superoxide Dismutase (SOD) activity↓	PPARα/RXRα/NF-κB	[69]
*Polygonum cuspidatum*	RSV	60	ISO-induced AMI	–	RSV(50/100 mg/kg, oral)	Oxidative stress ↓, myocardial necrosis ↓, interstitial edema ↓, neutrophil infiltration ↓	Cardiac Troponin T (cTnT)↑, LDH↑, AST↑, inflammatory cytokines (TNF-α, IL-1β, NF-κB, etc.) expression↓	–	[70]
Anti-fibrotic									
*Rabdosia rubescens (Hemsl.) H. Hararubescens*	Ori	–	LAD ligation	Ori/MCC950/CY-09	Saline	Neutrophil infiltration ↓, myocardial infarction area ↓, myocardial fibrosis ↓	IL-1β↓, IL-18↓, inflammatory infiltration of myocardial neutrophils↓, NOD-like receptor family, pyrin domain containing 3 (NLRP3) activation↓	–	[67]
*Salvia miltiorrhiza Bge*	STS	101	Clinical trial	Saline	STS (80 mg/day × 7 d)	Progressive left ventricular remodeling ↓, neutrophil degranulation and vesicle rupture ↓	Neutrophil elastase↓, myeloperoxidase↓, proteinase 3↓, Neutrophil Gelatinase-Associated Lipocalin (NGAL)↓, MMP-8↓, MMP-9↓, neutrophil infiltration and degranulation↓	–	[71]
*Astragalus membranaceus (Fisch.) Bunge*	AS-IV	–	LAD ligation	0.5% CMC (oral)	AS-IV (40 mg/kg, oral)	Myocardial fibrosis ↓, inflammatory infiltration ↓, myocardial injury ↓, cardiomyocyte hypertrophy ↓, cardiac remodeling ↓	Fibrosis markers (Collagen I, Collagen III, α-SMA, Fibronectin) expression↓	ROS/Caspase-1/GSDMD	[63]
*Epimedium grandiflorum*	ICA	100	Coronary ligation	Saline (2 mL/kg)	ICA (3–20 mg/kg)	Cardiac remodeling ↓, cardiomyocyte apoptosis rate ↓	Tissue Inhibitor of Metalloproteinases-1 (TIMP-1)↑, B-cell lymphoma 2 (Bcl-2)↑, MMP-9↓, Collagen I/III↓, CD147↓, BCL2-Associated X Protein (Bax)↓, caspase-3↓, cleaved caspase-3↓	CD147/MMP-9	[72]
Pro-angiogenic									
*Salvia miltiorrhiza Bge*	STS	56	LAD ligation	Saline (i.p.)	STS (20.8 mg/kg/day, i.p.)	Angiogenesis ↑, myocardial necrosis ↓, inflammatory cell infiltration ↓	CD31 + vessel density↓, Hypoxia-Inducible Factor-1α (HIF-1α) protein level↓, Vascular Endothelial Growth Factor (VEGF) level↓	–	[68]

**Table 2:** Neutrophil-targeting mechanisms of composite TCM prescriptions in AMI

Formula name	Composition	Traditional efficacy	Sample size	Study subject	Control treatment	Experimental treatment	Outcomes	Mechanisms	Pathway	Refs
Anti-inflammatory										
Tanhuo Decoction (THD)	Per dose: 9 g Coptidis Rhizoma, 5 g Rhei Radix et Rhizoma, 9 g Forsythia, 9 g Lophatherum gracile, 9 g Bile Arisaema	Heat-clearing, detoxifying, blood-activating	40	AMI patients	Standard Western medicine treatment (for 3 days)	Standard treatment + THD: 1 dose/day, divided into morning and evening doses (for 3 days)	Cardio-renal protection↑, metabolic regulation↑, inflammation↓, oxidative stress↓	CCR↑, neutrophil count↓, hs-CRP↓, pro-inflammatory proteins (Pentraxin 3 [PTX3], IL-18, IL-1, Cathepsin L1 [CTSL1], Tumor Necrosis Factor Receptor Superfamily Member 11A [TNFRSF11A], SPON2, CCL3, TRAIL-R2, Carcinoembryonic Antigen-Related Cell Adhesion Molecule 8 [CEACAM8]↓	PI3K-Akt, IL-17, MAPK, FoxO, JAK-STAT, HIF-1, Relaxin, VEGF, Wnt	[73]
Qingxin Jieyu Granule (QXJYG)	Astragalus membranaceus (Fisch.) Bunge, Salvia miltiorrhiza Bge. Bunge, Ligusticum chuanxiong Hort., Pogostemon cablin (Blanco) Benth., Coptis chinensis Franch	Blood-activating, toxin-resolving, stasis-dispersing, collateral-unblocking	10	LAD ligation-induced AMI model	Distilled water by gavage for 3 days	Oral gavage at 3 × human equivalent dose (6.9 g/kg/day)	Cardiac function↑, neutrophil recruitment/adhesion/migration to lesion site↓	Annexin A1 (ANXA1)↑, Formyl Peptide Receptor 2 (FPR2)↑, plasma histone-DNA levels↓, MPO↓, Citrullinated Histone H3 (CitH3)↓, NETs↓	ANXA1/FPR2	[74]
Kai-Xin-San (KXS)	Ginseng, Hoelen, Polygala, Acorus (3:3:2:2 ratio)	–	60	Isoproterenol-induced AMI model	Normal saline by gavage	1,785 mg/kg KXS by gavage for 14 days	Cardiomyocyte apoptosis↓, oxidative stress↓	Bcl-2↑, Bax↓, MMP-2↓, MMP-9↓	–	[75]
Anti-fibrotic										
Dan-shen Yin (DSY)	Salvia miltiorrhiza Bge., Santalum album L., Amomum villosum Lour	Blood-activating, stasis-resolving, qi-promoting, pain-alleviating	50	LAD ligation-induced AMI model	Nicorandil (1.35 mg/kg)	DSY high dose (4.02 g/kg), DSY low dose (2.01 g/kg)	Cardiac function↑, left ventricular ejection fraction↑, fractional shortening↑, myocardial injury↓, fibrosis↓, inflammation↓	IL-1β↓, IL-6↓, TNF-α)↓, COX2↓, Inducible Nitric Oxide Synthase (iNOS) mRNA↓, TGF-β↓, Collagen I↓, Collagen III↓, α-Smooth Muscle Actin (α-SMA)↓, MMP-2↓, MMP-9↓, serum Creatine Kinase-MB (CK-MB)↓, LDH↓	PI3K/AKT, MAPK, TGF-β	[76]

### Single-herb extracts in TCM

#### Anti-inflammatory effects of single-herb extracts in TCM

Substantial clinical evidence confirms that effective control of inflammatory responses significantly improves clinical outcomes in AMI patients and reduces the incidence of major adverse cardiovascular events (MACE). However, current anti-inflammatory therapies in Western medicine face multiple challenges. Existing drugs such as IL-1β monoclonal antibodies primarily target single inflammatory pathways, resulting in limited therapeutic efficacy while increasing infection risks and other serious adverse effects. The high treatment costs further restrict their clinical application [77]. Notably, the 2023 ESC guidelines for acute coronary syndrome management only provide a clear recommendation for low-dose colchicine (0.5 mg/day) in secondary prevention of coronary artery disease [78]. In this context, TCM demonstrates promising potential in AMI anti-inflammatory therapy due to its unique advantages of “holistic regulation and multi-target intervention”. Contemporary research reveals that single-herb TCM extracts may exert synergistic therapeutic effects through multiple mechanisms, including modulation of inflammatory networks and improvement of the myocardial microenvironment, thereby offering novel therapeutic strategies for inflammation management in AMI.

Astragaloside IV (AS-IV), one of the primary active components extracted from Astragalus membranaceus (Fisch.) Bunge, exhibits therapeutic potential against AMI. According to TCM theory, the anti-inflammatory effect of Astragalus originates from its primary function of ‘tonifying Qi’. The fundamental TCM principle that ‘when healthy Qi remains robust, pathogenic factors cannot invade’ emphasizes the crucial role of defensive Qi in protecting the body against external pathogens and preserving internal homeostasis. In cases where heart Qi deficiency leads to weakened defensive Qi, pathogenic factors may obstruct the cardiac vessels, resulting in pathological manifestations including inflammation. Unlike conventional anti-inflammatory approaches that directly eliminate heat and toxins, Astragalus, as a principal Qi-tonifying herb, achieves its anti-inflammatory effects by restoring Qi in the spleen, lungs, and heart. This mechanism strengthens the body’s innate regulatory and defensive systems, thereby targeting the underlying cause of inflammation and ultimately resolving the inflammatory condition [79]. Currently, although the “heart qi deficiency” syndrome has not yet formed a systematic and unified objective evaluation system, multiple studies have preliminarily revealed its intrinsic correlation with modern medical indicators. From the perspective of modern medicine, the pathological essence of “heart qi deficiency” is characterized by both the impairment of cardiac function indicators—primarily left ventricular ejection fraction (LVEF), left ventricular fractional shortening (LVFS), and B-type natriuretic peptide (BNP)—and the elevation of systemic inflammation levels represented by high-sensitivity C-reactive protein (hs-CRP). Specifically, LVEF, LVFS, and BNP, as key indicators for assessing cardiac pumping function and neuroendocrine activation, directly reflect the propelling and consolidating functions of heart qi. Meanwhile, hs-CRP, as an important inflammatory marker, reflects the pathological state of impaired heart qi and endogenous pathogenic turbidity when elevated. Together, these indicators construct an objective evaluation framework for “heart qi deficiency” syndrome from both functional and inflammatory dimensions [80, 81]. Previous studies have demonstrated that inhibition of signaling pathways such as GSDMD can provide neutrophil-mediated cardioprotection in AMI [31]. Pyroptosis, a caspase-1-dependent programmed cell death process accompanied by massive release of proinflammatory cytokines, contributes significantly to systemic inflammation, while the NLRP3 inflammasome is known to promote caspase-1 activation [82]. In their investigation, Zhang et al. [63] established an AMI model through ligation of the left anterior descending coronary artery in mice and employed multiple analytical techniques including immunofluorescence, flow cytometry, and immunohistochemistry to examine the effects of AS-IV on AMI-induced myocardial fibrosis and cardiac remodeling. Results demonstrated that AS-IV significantly restored LVEF and LVFS at the core cardiac functional level, directly reflecting its effect of replenishing heart qi and enhancing cardiac pumping capacity. In terms of inflammatory regulation, AS-IV markedly suppressed myocardial neutrophil infiltration, as evidenced by reduced MPO expression, decreased ROS levels, and subsequent inhibition of NLRP3 inflammasome activation and cardiomyocyte pyroptosis. Ultimately, AS-IV alleviated cardiac dysfunction and inflammatory responses by modulating the ROS/Caspase-1/GSDMD signaling pathway. This study systematically elucidates the experimental basis for Astragalus membranaceus in ameliorating "heart qi deficiency" syndrome through its "reinforcing qi and consolidating the defensive exterior" mechanism, targeting both cardiac functional improvement and inflammatory suppression. Curcumin, a component of the traditional medicine Curcuma longa L., has anti-inflammatory, antimicrobial, anti-inflammatory and anti-aging abilities [83]. Boarescu et al. [64] administered curcumin nanoparticles (CCNP) to white rats by intragastrical administration at three different doses and evaluated the antioxidant, anti-inflammatory and cardioprotective effects in each group. The results showed that CCNP had cardioprotective effects, which were attributed to their ability to enhance the antioxidant response and reduce the serum levels of pro-inflammatory cytokines and MMP expression in myocardial ischemia. Compared to conventional CC, CCNP demonstrate enhanced targeted delivery of curcumin compounds to the cardiac endocardial layer, thereby exerting more pronounced cardioprotective effects in myocardial tissue. These nanoparticles exhibit superior efficacy in preventing elevated levels of proteolytic enzymes MMP-9 and MMP-2, reducing myocardial necrosis, attenuating interstitial edema and neutrophil infiltration, while significantly lowering pro-inflammatory cytokine levels (particularly TNF-α), collectively contributing to their potent anti-oxidative and therapeutic effects against AMI pathogenesis. Resveratrol (RSV) isolated from the root of Polygonum cuspidatum is a natural plant antitoxin with anti-inflammatory effects. Sun et al. [70] used conventional RSV as a control to observe the effect of poly (lactic-co-glycolic) acid nanoparticle loaded with RSV PLGA NPs on MI in rats by assessing the antioxidant, anti-inflammatory, and cardioprotective effects of the groups. The final results indicated that compared with RSV, RSV PLGA NPs more effectively prevented myocardial necrosis, reduced interstitial edema and neutrophil infiltration, and suppressed the expression of inflammatory factors such as TNF-α, IL-1β, and NF-kB after MI, consequently limiting cardiac injury after MI.

Poria cocos (Schw.) Wolf polysaccharide (PCP), one of the primary active components of Poria cocos (Schw.) Wolf, has demonstrated anti-inflammatory, antitumor, and antioxidant properties. In a study by Xie et al. [65], a AMI rat model was established to investigate the effects and mechanisms of PCP on cardiomyocyte apoptosis in AMI using Western blot and ELISA assays with saline as control. The results showed that PCP alleviated cardiomyocyte swelling and neutrophil infiltration. Further research revealed that PCP reduced serum levels of proinflammatory factors IL-1β and IL-18, inhibited neutrophil adhesion and oxygen free radical production, thereby improving tissue edema and microcirculatory dysfunction. The cardioprotective effects of PCP against myocardial tissue damage and apoptosis may be mediated through suppression of the Rho-ROCK signaling pathway, leading to attenuated oxidative stress and inflammatory responses.

Colchicine, derived from the medicinal herb Colchicum autumnale L., has been employed in treating various cardiovascular diseases including AMI due to its unique anti-inflammatory properties [84]. Using a permanent left anterior descending coronary artery ligation model in mice, Li et al. [14] demonstrated that colchicine exerts cardioprotective effects in AMI by inhibiting NET formation through reduction of NOX2/ROS production and Ca2 + influx, as evidenced by echocardiography and immunofluorescence assays, ultimately attenuating post-AMI remodeling and cardiac inflammation.

Oridonin (Ori), a natural terpenoid compound isolated from Rabdosia rubescens (Hemsl.) H. Hararubescens, has been shown to modulate post-AMI inflammatory responses. Following AMI, specific alarmins released by neutrophils interact with TLR-4 on newly recruited neutrophils, activating the NLRP3 inflammasome [23]. Gao et al. [67] found that Ori reduced inflammatory infiltration of neutrophils in myocardial tissue and suppressed expression of proinflammatory factors IL-1β and IL-18 through inhibition of NLRP3 activation.

Sodium tanshinone IIA sulfonate (STS), an active extract from Salvia miltiorrhiza Bge., exhibits significant anti-inflammatory activity. Zhang et al. [68] investigated the role of STS in post-AMI ventricular remodeling using a murine AMI model with saline control. STS treatment improved cardiac function, reduced scar size, and decreased levels of α-smooth muscle actin, collagen I/III, inflammatory cell infiltration, plasma LDH, high mobility group box 1, interleukin-1β, tumor necrosis factor-α, and their protein expressions. Both tanshinones and phenolic acids, the major active components of Salvia miltiorrhiza Bge., demonstrate multiple pharmacological effects including microcirculation improvement, anti-atherosclerotic, anti-inflammatory, and antitumor activities. In a systematic comparison of sweated versus non-sweated Salvia extracts using a rat AMI model, Shan et al. [69] found that both extracts significantly reduced ST-segment elevation, inflammatory cell infiltration, and infarct size while downregulating inflammatory factors and oxidative stress. Mechanistic studies revealed that these anti-inflammatory effects were closely associated with modulation of the PPARα/RXRα/NF-κB signaling pathway. Notably, the sweating process enhanced the biological activity of Salvia extracts, with sweated extracts demonstrating superior efficacy and stronger anti-inflammatory effects from tanshinones compared to phenolic acids, providing important theoretical basis for optimizing Salvia processing techniques and developing potent anti-inflammatory drugs.

Resveratrol (RSV), a natural phytoalexin with anti-inflammatory properties isolated from Polygonum cuspidatum roots, was investigated by Sun et al. [70] using poly (lactic-co-glycolic) acid nanoparticles loaded with resveratrol (RSV PLGA NPs) in a rat MI model. Compared to conventional RSV, RSV PLGA NPs demonstrated enhanced protection against myocardial necrosis, interstitial edema, neutrophil infiltration, and expression of inflammatory factors (TNF-α, IL-1β, NF-κB), thereby more effectively limiting post-MI cardiac injury.

#### Anti-fibrotic effects of single-herb extracts

Post-AMI fibrosis is a critical pathological process driving ventricular remodeling, with emerging evidence highlighting the pivotal role of neutrophils and their secreted inflammatory mediators. Recent studies have identified multiple natural compounds (e.g., Ori, STS, AS-IV) that exert significant anti-fibrotic effects through distinct mechanisms.

Ori, the primary active constituent of Rabdosia rubescens (Hemsl.) H. Hararubescens, demonstrates broad anti-fibrotic activity across various cell types and organs, including human gingival fibroblasts and fibroblast-like synoviocytes [85]. In a murine AMI model, Gao et al. [67] employed inflammatory marker analysis and immunohistochemistry to evaluate Ori’s effects on myocardial inflammatory factors and fibrosis markers. Their findings revealed that Ori reduced neutrophil-mediated inflammatory infiltration and suppressed myocardial expression of IL-1β and IL-18 through NLRP3 inflammasome inhibition, ultimately improving cardiac function, limiting infarct expansion, and attenuating myocardial fibrosis.

STS, an active extract from Salvia miltiorrhiza Bge., modulates cardiac fibroblast function through multiple pathways: it inhibits type I collagen synthesis via angiotensin receptor pathways while enhancing neoclastin deposition through PKA/CREB activation. Beyond its cardioprotective effects against cardiomyocyte hypertrophy, STS suppresses cardiac fibroblast proliferation and collagen production, thereby improving coronary circulation and preventing thrombosis. Mechanistic studies confirm that STS upregulates miR-205-3p and miR-618 while downregulating TGF-β1, effectively reducing pro-fibrotic factor expression to ameliorate post-MI fibrosis and ventricular remodeling [86, 87]. In a clinical study of 101 STEMI patients with successful reperfusion, Mao et al. [71] compared STS intravenous therapy against saline control using echocardiography and proteomic analysis. STS treatment significantly reduced plasma levels of neutrophil-derived granular components (elastase, myeloperoxidase, proteinase 3, NGAL, MMP-8/9), inhibited neutrophil infiltration and degranulation, and attenuated adverse ventricular remodeling. These results suggest that adjunctive STS therapy mitigates early release of matrix degradation products that recruit inflammatory factors, thereby slowing left ventricular remodeling and improving clinical outcomes—providing evidence-based rationale for optimizing AMI treatment protocols.

According to TCM theory, the anti-fibrotic effect of Astragalus membranaceus (Fisch.) Bunge is fundamentally a molecular manifestation of its "Qi-tonifying and blood-activating" efficacy. This mechanism originates from the initial pathological link of Heart-Qi deficiency: Qi insufficiency leads to impaired circulatory dynamics, resulting in the accumulation of pathological byproducts such as Qi stagnation, phlegm turbidity, and blood stasis, which ultimately drive aberrant tissue remodeling. Functionally, Astragalus membranaceus (Fisch.) Bunge replenishes Heart-Qi to modulate key signaling pathways (e.g., PI3K/AKT, NF-κB), thereby suppressing oxidative stress and inflammatory responses—the core drivers of fibrosis. Concurrently, it regulates glucolipid metabolism, ion homeostasis, and hemorheological properties at the material level, providing a metabolic foundation for tissue repair. This multi-level regulation collectively interrupts the “phlegm-stasis interaction” pathology, exemplifying the therapeutic logic of “tonifying Qi to eliminate pathogens and activating blood to unblock vessels” [79]. The active compound AS-IV further targets fibrotic progression through cellular biological pathways, including inhibition of extracellular matrix synthesis and blockade of fibroblast transformation [88]. Zhang et al. [63] demonstrated that AS-IV downregulates the ROS/NLRP3/GSDMD pathway, suppressing expression of NLRP3 inflammasome components (cleaved caspase-1, IL-1β, IL-18, GSDMD-N) to exert multifaceted cardioprotection: reducing myocardial injury, pathological hypertrophy, inflammatory infiltration, and fibrosis while improving post-AMI ventricular remodeling.

Epimedium grandiflorum exhibits notable anti-fibrotic activity attributable to its bioactive flavonoid icariin (ICA) [89]. Shi et al. [72] elucidated ICA’s protective mechanisms against post-AMI cardiac remodeling, showing that ICA reduces cardiomyocyte apoptosis through CD147/MMP-9 pathway inhibition and MMP-9 downregulation. This significantly decreases neutrophil activation, myocardial fibrosis, and pathological hypertrophy, ultimately improving ventricular remodeling.

#### Pro-angiogenic effects of single-herb extracts

Emerging evidence highlights therapeutic angiogenesis as a critical strategy for cardiac repair post-AMI. Previous studies demonstrate that STS, an active extract from Salvia miltiorrhiza Bge., enhances vascular endothelial integrity and promotes angiogenesis [90]. In a murine AMI model induced by left anterior descending artery ligation, Zhang et al. [68] systematically investigated STS’s role in ventricular remodeling using saline controls. Comprehensive assessments including gechocardiography for cardiac function, trichrome staining and immunoblotting for fibrosis markers, ELISA/immunohistochemistry for necrosis/inflammation, and CD31 + immunohistochemistry for angiogenesis revealed that STS treatment significantly increased microvessel density, hypoxia-inducible factor-1α (HIF-1α) expression, and vascular endothelial growth factor (VEGF) levels, confirming its potent pro-angiogenic properties.

### Compound formulations

#### Anti-inflammatory effects of compound formulations

Tanhuo Decoction (THD), a multi-herb formulation with heat-clearing and blood-activating properties, demonstrates multifaceted therapeutic value in acute cardiovascular events. A randomized controlled trial by Guo et al. [73] involving 40 AMI patients (aged 18–80 with type I AMI onset < 12 h and high-sensitivity C-reactive protein [hs-CRP] ≥ 3 mg/L) compared standard care (aspirin, clopidogrel, enoxaparin, atorvastatin) versus standard care plus THD (200 mL/day divided doses for 3 days). THD significantly reduced hs-CRP and oxidative stress while improving uric acid metabolism, demonstrating concurrent cardiorenal protection through multi-target mechanisms.

Qingxin Jieyu Granule (QXJYG), developed by cardiologist Chen Keji based on "blood stasis-toxin" theory. This Theory postulates that coronary artery disease originates from chronic blood stasis, whereas acute coronary syndromes (e.g., AMI) manifest through toxin-triggered stasis exacerbation. This paradigm bridges TCM and modern cardiology by demonstrating how acute pathological events—including plaque rupture and inflammatory cascades—exhibit striking parallels with TCM-defined toxin pathogens characteristics: abrupt onset and severe endothelial disruption. Qi et al. systematically investigated the therapeutic effects and molecular mechanisms of QXJYG in AMI using a rat model [74]. Collectively, these results establish that QXJYG improved cardiac function by activating the ANXA1/FPR2 pathway to inhibit NET formation. Network pharmacology identified FPR2 as the pivotal target, where ANXA1-FPR2 binding suppressed neutrophil chemotaxis/adhesion, modulating inflammatory resolution.

Kai-Xin-San (KXS), a four-herb antidepressant formula (3:3:2:2 ratio of ginseng, hoelen, polygala, and acorus), exhibited dual cardioprotective/antidepressant effects in a novel AMI-depression rat model [75]. KXS downregulated MMP-2/9 expression and restored Bcl-2/Bax balance, attenuating cardiomyocyte apoptosis and oxidative stress.

#### Anti-fibrotic effects of compound formulations

Dan-shen Yin (DSY), a Qing-dynasty formula for cardiovascular diseases, was investigated by Gao et al. [76] using pharmacodynamic assays and multi-omics integration. DSY ameliorated myocardial fibrosis by modulating TGF-β-dependent PI3K/AKT, MAPK, and Smad pathways, reducing extracellular matrix deposition and focal adhesion formation while suppressing ECM-receptor interactions and inflammation.

## Clinical applications

Recent advances in neutrophil-targeted therapies for cardiovascular diseases have yielded progress across three key domains: (1) mechanistic exploration of conventional drugs and development of novel targeted agents, (2) innovative integration of nanomedicine with neutrophil modulation, and (3) precision diagnostics leveraging neutrophil multi-omics profiling. TCM exhibits unique potential for synergistic integration: Mechanistically, its "multi-target, systemic regulation" approach complements existing therapies by filling pharmacological gaps while minimizing adverse effects. Technologically, nanoparticle-based delivery systems enhance the targeting efficiency of bioactive TCM compounds to neutrophils. Diagnostically, correlating neutrophil molecular subtypes with TCM syndrome differentiation patterns enables personalized therapeutic strategies. This interdisciplinary convergence not only strengthens the scientific rationale for TCM-mediated neutrophil regulation but also pioneers precision medicine approaches for AMI through integrative medicine paradigms.

### Advances in neutrophil-targeted drug development

Current therapeutic strategies targeting neutrophil-associated inflammation have progressed in three aspects: optimized use of clinical-stage drugs, development of novel agents, and identification of innovative targets. Clinically, the anti-inflammatory mechanisms of P2Y12 receptor antagonists (e.g., cangrelor) have been elucidated, while emerging biologics like complement modulators demonstrate therapeutic potential through neutrophil regulation. Furthermore, discoveries such as the NR4A orphan nuclear receptors provide novel targets for precisely modulating neutrophil-driven inflammatory cascades, marking a paradigm shift from purely antithrombotic to multi-target immunomodulatory therapies.

A prospective open-label randomized study demonstrated that early cangrelor administration in STEMI patients undergoing Percutaneous Coronary Intervention (PCI) not only enhanced platelet inhibition but also significantly reduced circulating inflammatory cells, proinflammatory cytokines, and neutrophil elastase-myeloperoxidase complexes (surrogates of NETosis), thereby attenuating systemic inflammation and myocardial injury [91].

The serine protease inhibitor C1 esterase inhibitor (C1INH) exhibited cardioprotective effects in rat AMI models by modulating apoptotic (Bcl-2/Bax ratio) and inflammatory pathways (neutrophil infiltration suppression), ultimately reducing infarct size [92].

NR4A3, a member of the NR4A orphan nuclear receptor family, emerged as a promising therapeutic target. Jiang et al. [93] showed that NR4A3 overexpression in murine AMI models suppressed neutrophil infiltration through dual mechanisms: inhibiting NF-κB/IκB signaling while activating JAK2-STAT3, thereby improving ventricular function. Phosphatidylserines (PS), bioactive phospholipids with neuroprotective properties, demonstrated cardioprotection in murine AMI models by selectively inhibiting neutrophil activation (e.g., IL-1β downregulation) without compromising physiological repair processes, suggesting clinical potential for reducing infarct size and perioperative myocardial protection [94].

### Integration of nanotechnology in therapeutic strategies

Emerging neutrophil-targeted nanotechnologies in cardiovascular medicine have developed along two innovative trajectories: (1) precision modulation of neutrophil activation states through targeted nanoparticle formulations, and (2) bioengineered drug delivery platforms leveraging neutrophil membrane biomimetics or live-cell engineering. While clinical translation for AMI treatment remains exploratory, these approaches demonstrate significant translational value by addressing critical challenges in inflammatory regulation.

Cell membrane-coated nanotechnology represents a breakthrough in targeted delivery systems, utilizing native cell membranes to encapsulate nanoparticles [95]. Neutrophils—the most abundant immune cells infiltrating infarcted myocardium—exhibit unique biological properties through upregulated adhesion molecules that mediate endothelial interactions. Their membranes provide ideal coating materials due to inherent biocompatibility and targeting capabilities, enabling prolonged circulation while maintaining immune quiescence. Neutrophil membrane-coated therapeutic nanoparticles thus achieve enhanced tissue specificity and pharmacokinetics.

Han et al. [96] developed a biomimetic nanoparticle system (NM-NPIL-5) delivering interleukin-5 (IL-5), a pro-angiogenic cytokine critical for cardiac repair. Combining in vitro (HUVEC models) and in vivo (murine LAD ligation) studies, this platform exploited neutrophil membranes’ chemotactic properties to precisely target infarcted myocardium, simultaneously promoting angiogenesis and suppressing inflammation without off-target effects.

Similarly, Wang et al. [97] engineered an anti-pyroptotic nanoplatform (NM@PDA@PU) featuring neutrophil membrane-coated polydopamine nanoparticles loaded with puerarin. This system mitigated post-AMI injury through dual mechanisms: direct inhibition of the NLRP3-caspase-1-IL-1β/IL-18 pyroptosis pathway and polarization of macrophages toward anti-inflammatory M2 phenotypes, disrupting the proinflammatory cascade between macrophages and pyroptotic cardiomyocytes.

Despite promising preclinical results, clinical translation of neutrophil-targeted therapies (e.g., S100A8/A9 inhibitor ABR2575, β1-adrenergic antagonist metoprolol, anti-Ly6G antibody-mediated neutrophil depletion) has been limited by suboptimal pharmacokinetics. Nanotechnology addresses these challenges by precisely modulating drug distribution and targeting efficiency [98]. Su et al. [99] developed a catalytic immunomodulatory nanocomplex encapsulating hydrophilic ABR2575 within nanoemulsions, which selectively targeted activated proinflammatory neutrophils in infarct zones. This approach effectively blocked neutrophil-S100A8/A9 signaling, reducing oxidative stress, infarct size, and functional impairment—demonstrating the potential of engineered nanomaterials for precision AMI therapeutics.

### Predictive and stratification studies of AMI patients based on neutrophil multi-omics data

The current diagnosis of AMI primarily relies on characteristic abnormalities in cardiac biomarkers such as cardiac troponin (cTn) to identify myocardial injury. However, existing biomarkers exhibit significant limitations—while elevated levels of markers like cTn reflect myocardial damage, they cannot effectively distinguish AMI from other pathophysiological conditions (e.g., non-acute coronary syndromes [ACS] or chronic cardiac diseases), indicating they possess organ specificity (cardiac specificity) but lack disease specificity [100]. These diagnostic shortcomings in clinical practice highlight the urgent need to develop novel biomarkers that combine excellent diagnostic sensitivity with high specificity.

Recent research advances demonstrate that neutrophil-derived indicators show exceptional clinical potential, ranging from traditional inflammatory ratios (e.g., neutrophil-to-lymphocyte ratio [NLR], neutrophil-to-albumin ratio [NPAR]) to cutting-edge molecular signatures and genetic regulatory networks. As summarized in Table 3, these parameters demonstrate significant translational value in early risk stratification, precise prognosis assessment, and personalized treatment decision-making for AMI.

**Table 3:** Predictive and stratification studies of AMI patients based on neutrophil multi-omics data

Biomarker	Study Population	Objective	Methodology	Key findings	References
Neutrophil-to-serum iron ratio (N/SI)	263 AMI patients	Identify potential biomarkers for AMI and Gensini score correlation	Gensini scoring, ROC curve analysis	N/SI showed the highest AUC among inflammatory biomarkers for AMI diagnosis and correlated with Gensini scores	[101]
Neutrophil-to-lymphocyte ratio (NLR)	1,550 elderly AMI patients (≥ 60 years)	Predict in-hospital mortality risk in elderly AMI patients	Retrospective observational study, ROC analysis, Cox regression	NLR significantly correlated with in-hospital mortality (P < 0.001); NLR > 6.69 independently predicted short-term adverse outcomes	[102]
NLR	798 critically ill AMI patients	Predict all-cause mortality in severe AMI	Cox regression, subgroup analysis	Admission NLR independently predicted 180-day and 365-day all-cause mortality (P < 0.05)	[103]
Neutrophil count	3,062 AMI patients (76% White, 24% minority)	Examine ethnic differences in neutrophil count-prognosis association	Cohort study	Minority patients had lower baseline neutrophil counts but stronger mortality associations at elevated levels (P < 0.05)	[104]
NLR	782 critically ill AMI patients	Predict prognosis in critical AMI	Retrospective cohort, Cox regression	NLR independently predicted 1-year mortality, 90-day mortality, and in-hospital death	[106]
NLR	1,289 NSTEMI and 1,329 STEMI patients	Compare NLR’s predictive value for NSTEMI vs STEMI short-term prognosis	Single-center retrospective observational study	NLR better predicted NSTEMI in-hospital mortality (AUC = 0.746) than STEMI (AUC = 0.621); influenced by age, creatinine, LDL-C, diabetes, smoking	[107]
NPAR, MLR	76 AMI patients with free wall rupture (FWR)	Predict FWR	Multivariate logistic regression, ROC analysis	NPAR (AUC = 0.811, P < 0.001) and MLR (AUC = 0.778, P < 0.001) were strong FWR predictors	[108]
NMLR	2259 AMI patients	Predict in-hospital mortality	Cox regression, ROC analysis	Elevated admission NMLR independently predicted in-hospital mortality (P < 0.05)	[109]
NPR	664 AMI patients	Identify high-risk patients for in-hospital mortality	Univariate analysis, ROC curves	NPR independently predicted in-hospital mortality (P < 0.001)	[110]
NLR	1000 CAD patients	Predict in-hospital mortality in elderly AMI	Retrospective cohort, GRACE score, ROC analysis	Elevated NLR potentially predicted in-hospital mortality in elderly AMI patients (P < 0.05)	[111]

AUC, area under the curve; NPAR, neutrophil-to-albumin ratio; MLR, monocyte-to-lymphocyte ratio; NMLR, neutrophil-monocyte-to-lymphocyte ratio; NPR, neutrophil-to-platelet ratio

All statistical significances maintain original P-values from source studies.

In their evaluation of 263 AMI patients, Ma et al. [101] found significantly elevated neutrophil-to-serum iron (N/SI) ratios that positively correlated with coronary stenosis severity (Gensini scores). Multivariate analysis confirmed N/SI as an independent risk factor for AMI, establishing it as a novel inflammatory marker for assessing coronary lesions.

Chen et al. [102] conducted a retrospective observational study of 1,550 elderly (age ≥ 60 years) AMI patients from the Second Affiliated Hospital of Dalian Medical University. The results demonstrated a significant association between NLR and in-hospital mortality risk in elderly AMI patients, with multivariate regression analysis confirming that high NLR (> 6.69) serves as an independent predictor of short-term adverse outcomes in this population.

Lin et al. [103] performed a retrospective cohort study of 798 critically ill AMI patients using intensive care database records, systematically examining the prognostic relationship between admission NPAR and all-cause mortality at different time points (30/90/180/365 days). The study revealed that higher admission NPAR showed significant independent correlations with 180-day and 365-day all-cause mortality. These findings establish NPAR as an effective long-term prognostic indicator for critically ill AMI patients, with cost-effective measurement methods that demonstrate important clinical utility in resource-limited healthcare settings, providing an objective basis for risk stratification and precision interventions.

Sadler et al. [104] conducted a cohort study of 3,062 AMI patients (76% White, 24% ethnic minorities), revealing that ethnic minority patients had lower baseline neutrophil counts compared to White patients. Importantly, elevated neutrophil levels showed stronger mortality risk associations in minority patients, with distinct predictive thresholds across ethnic groups. This study emphasizes how understanding ethnic-specific differences in post-AMI inflammatory responses carries critical clinical significance for achieving precise prognosis assessment and personalized patient management.

Through analysis of AMI patient blood gene data and machine learning approaches, Zhu et al. [105] developed a neutrophil programmed cell death signature (NPCDS) model that successfully distinguished between different myocardial infarction types. Subsequent mouse experiments and computational modeling elucidated the biological mechanisms underlying this model, while genetic analysis and drug simulations validated its clinical potential. The study identified MDM2 as a central regulator in the NPCDS network that serves dual roles as both a disease prediction marker and therapeutic target, providing novel strategies for precision diagnosis and treatment of AMI.

## Conclusions

AMI represents a severe cardiovascular condition with significant clinical implications. Despite its clinical importance, current therapeutic approaches for AMI remain limited, highlighting the critical need to investigate the role of neutrophils in AMI pathogenesis. As the first immune cells recruited to infarcted myocardium and key players throughout AMI progression, neutrophils have emerged as promising therapeutic targets for myocardial infarction management. This review systematically examines neutrophil involvement in AMI through three key aspects: origin and subtypes, stage-specific functional characteristics, and molecular mechanisms. With advancing research technologies, neutrophil classification has evolved from the simplistic N1/N2/N3 paradigm to more sophisticated subpopulations characterized by scRNA-seq, revealing their dynamic changes during AMI. Neutrophil activity in AMI progresses through two distinct phases: the initial inflammatory phase and subsequent repair/proliferation/maturation phase. Pre-existing neutrophils from BM are chemotactically recruited to infarct zones post-AMI, initially exerting pro-inflammatory effects before transitioning to anti-inflammatory phenotypes that facilitate tissue remodeling, wound healing, and scar formation. Research demonstrates neutrophils’ dual roles in both inflammatory responses and myocardial fibrosis, while also influencing angiogenesis, cardiac electrical conduction, and thrombus formation.

TCM has demonstrated notable safety and efficacy in AMI management, supported by extensive clinical applications. The multi-component, multi-target nature of TCM formulations necessitates their systematic investigation. Our review of recent studies reveals that TCM interventions—including single-herb extracts and compound formulations—primarily modulate neutrophil activity in AMI through three mechanisms: anti-inflammatory effects (the most extensively studied), anti-fibrotic actions, and pro-angiogenic properties. Notable investigated compounds include astragaloside IV, curcumin, and resveratrol, which show significant neutrophil-regulating effects. However, several research limitations persist: (1) The current body of evidence is constrained by a limited number of studies, which frequently utilize small sample sizes (e.g., n = 6–10 in animal experiments) and exhibit inadequate control over experimental variables. The reliability of findings is further compromised by significant heterogeneity in the extraction methodologies for herbal compounds, such as variations in ethanol concentration (ranging from 50 to 95%), sonication duration (varying between 15 and 60 min), and the employment of different techniques (e.g., water decoction versus ethanol reflux). Consequently, large-scale experimental data and robust pre-clinical studies are imperative to validate these results and assess their potential for future clinical translation; (2) Most studies employ simplistic experimental designs with limited controls, hindering precise target/pathway identification; (3) The vast majority of potentially effective TCM compounds remain unexplored. In the field of traditional Chinese medicine, AMI is often classified under the syndrome of "chest obstruction," and classical formulas such as Xuefu Zhuyu Decoction, Gualou Xiebai Baijiu Decoction, and Shenfu Decoction have demonstrated certain therapeutic efficacy. Future research should prioritize selecting specific herbs or herb pairs from these classical formulas and employ modern technologies to elucidate their mechanisms of action. Specifically, studies could focus on validating whether Xuefu Zhuyu Decoction modulates the function of the SigFᴴᴵ neutrophil subset to inhibit NETs formation and attenuate inflammatory responses. Concurrently, systematic evaluation of the effects of Gualou Xiebai Baijiu Decoction on neutrophil polarization—such as the N1/N2 phenotypic switch—and myocardial fibrosis would help clarify the immunological basis underlying its traditional action of "unblocking yang and dissipating nodules.

Following a summary of the current research status on TCM for regulating neutrophils in the treatment of AMI, it is necessary to further explore the latest advances in neutrophil-targeted strategies within AMI clinical management. This endeavor aims to provide new insights for TCM-based AMI treatment and related mechanistic investigations. Currently, neutrophil-targeted therapy has evolved into a multidimensional system encompassing drug development, nanotechnology, and precision medicine, demonstrating a clear trajectory from basic research toward clinical translation. In the realm of pharmaceutical development, beyond conventional antiplatelet agents, novel drugs targeting pathways such as the complement system and nuclear receptors are continuously emerging. Concurrently, numerous natural medicinal compounds have been confirmed to exert cardioprotective effects by modulating neutrophil function, indicating promising therapeutic prospects.

However, despite the demonstrated potential of TCM in multi-target synergistic regulation of neutrophils, key challenges remain for its clinical translation. Current research is predominantly confined to animal studies and in vitro models, lacking robust support from large-scale, multicenter, randomized controlled clinical trials to validate its efficacy and safety. Furthermore, the complex composition of TCM formulas presents hurdles: the pharmacokinetics, bioavailability, and in vivo targets of their active constituents require further clarification, and standardized preparation processes along with quality control systems need enhancement.

Notably, nanotechnology has introduced significant breakthroughs in neutrophil-targeted therapy. Biomimetic nanocarrier systems not only enhance drug targeting efficiency but also achieve synergistic anti-inflammatory and pro-repair effects, offering a potential strategy to overcome the limitations of poor delivery efficiency and targeting specificity associated with TCM components. In diagnostics, novel biomarkers and predictive models based on neutrophil multi-omics data have substantially improved risk stratification and prognostic assessment for AMI. These advancements provide expanded options for AMI clinical management and lay a foundation for personalized precision medicine.

Future research should focus on overcoming the current translational bottlenecks. This can be achieved by strengthening the bridge between basic and clinical research, promoting high-quality preclinical and clinical studies, and integrating modern technologies such as multi-omics analysis and nano-drug delivery systems to deepen the understanding of the mechanisms by which TCM regulates neutrophils. Accelerating the clinical translation process and ultimately fostering multidisciplinary collaboration will be crucial for advancing the widespread clinical application of neutrophil-targeted therapies in AMI.

## Supplementary Information

Supplementary Material 1

## Data Availability

No datasets were generated or analysed during the current study.

## Abbreviations

ACS: Acute Coronary Syndrome
AMI: Acute Myocardial Infarction
ANXA1: Annexin A1
Arg1: Arginase-1
AS-IV: Astragaloside IV
AST: Aspartate Aminotransferase
AUC: Area Under the Curve
Bax: BCL2-Associated X Protein
Bcl-2: B-cell lymphoma 2
BM: Bone Marrow
BNP: B-type Natriuretic Peptide
C5a: Complement component 5a
CAD: Coronary Artery Disease
CC: Curcumin
CCNP: Curcumin Nanoparticles
Ccl: C-C motif chemokine ligand
CEACAM8: Carcinoembryonic Antigen-Related Cell Adhesion Molecule 8
CitH3: Citrullinated Histone H3
CK-MB: Creatine Kinase-MB
C1INH: C1 esterase inhibitor
CMC: Carboxymethyl Cellulose
CRP: C-Reactive Protein
CSF: Colony Stimulating Factor
CSF1: Colony Stimulating Factor 1
CSF3R: Colony Stimulating Factor 3 Receptor
CTSL1: Cathepsin L1
cTn: Cardiac Troponin
cTnT: Cardiac Troponin T
CXCL: C-X-C Motif Chemokine Ligand
CXCR: C-X-C Motif Chemokine Receptor
CXCR2: C-X-C Motif Chemokine Receptor 2
DAMPs: Damage-Associated Molecular Patterns
DNase: Deoxyribonuclease
dsDNA: double-stranded DNA
DSY: Dan-shen Yin
ECG: Electrocardiogram
ECM: Extracellular Matrix
ELISA: Enzyme-Linked Immunosorbent Assay
EPC: Endothelial Progenitor Cell
ESC: European Society of Cardiology
FPR2: Formyl Peptide Receptor 2
FoxO: Forkhead box O
FUNDC1: FUN14 domain-containing 1
FWR: Free Wall Rupture
G-CSF: Granulocyte Colony-Stimulating Factor
GSDMD: Gasdermin D
HIF-1α: Hypoxia-Inducible Factor-1α
HMGB1: High Mobility Group Box 1
HSC: Hematopoietic Stem Cell
hs-CRP: high-sensitivity C-Reactive Protein
HUVEC: Human Umbilical Vein Endothelial Cell
I/R: Ischemia-Reperfusion
ICA: Icariin
ICAM1: Intercellular Adhesion Molecule 1
IL: Interleukin
IL-1β: Interleukin-1β
IL1R2: IL1R2
iNOS: Inducible Nitric Oxide Synthase
i.p.: Intraperitoneal
ISGs: Interferon-Stimulated Genes
JAK-STAT: Janus Kinase-Signal Transducer and Activator of Transcription
KXS: Kai-Xin-San
LAD: Left Anterior Descending (coronary artery)
LCN2: Lipocalin-2
LDH: Lactate Dehydrogenase
LDL-C: Low-Density Lipoprotein Cholesterol
LVEF: Left Ventricular Ejection Fraction
LVFS: Left Ventricular Fractional Shortening
Ly6G: Lymphocyte antigen 6 complex, locus G
MACE: Major Adverse Cardiovascular Events
MAPK: Mitogen-Activated Protein Kinase
MDA: Malondialdehyde
Mif: Macrophage Migration Inhibitory Factor
MI: Myocardial Infarction
miRNA: MicroRNA
MLR: Monocyte-to-Lymphocyte Ratio
MMP: Matrix Metalloproteinase
MMP-9: Matrix Metalloproteinase-9
MPO: Myeloperoxidase
MVO: Microvascular Obstruction
NETs: Neutrophil Extracellular Traps
NETosis: Neutrophil Extracellular Trap-associated cell death
NF-κB: Nuclear Factor kappa-light-chain-enhancer of activated B cells
NGAL: Neutrophil Gelatinase-Associated Lipocalin
NLR: Neutrophil-to-Lymphocyte Ratio
NLRP3: NOD-like receptor family pyrin domain-containing 3 inflammasome
NMLR: (Neutrophil+Monocyte)-to-Lymphocyte Ratio
NO: Nitrogen Oxides
NOX2: NADPH Oxidase 2
NPAR: Neutrophil Percentage-to-Albumin Ratio
NPCDS: Neutrophil Programmed Cell Death Signature
NPR: Neutrophil-to-Platelet Ratio
N/SI: Neutrophil-to-Serum Iron ratio
NSFC: National Natural Science Foundation of China
NSTEMI: Non-ST-Segment Elevation Myocardial Infarction
Ori: Oridonin
PCI: Percutaneous Coronary Intervention
PCP: Poria cocos Polysaccharide
PI3K: Phosphoinositide 3-kinase
PLGA: Poly(lactic-co-glycolic) acid
polyP: Polyphosphate
PPAR: Peroxisome Proliferator-Activated Receptor
PS: Phosphatidylserines
PTX3: Pentraxin 3
QXJYG: Qingxin Jieyu Granule
Rho-ROCK: Rho-associated protein kinase
ROC: Receiver Operating Characteristic
ROS: Reactive Oxygen Species
RSV: Resveratrol
RXRα: Retinoid X Receptor alpha
s.c.: Subcutaneous
scRNA-seq: Single-Cell RNA Sequencing
SigF / SiglecF: Sialic acid-binding Ig-like lectin F
SOD: Superoxide Dismutase
Sod2: Superoxide Dismutase 2
SPON2: Spondin-2
STAT: Signal Transducer and Activator of Transcription
STEMI: ST-Segment Elevation Myocardial Infarction
STS: Sodium Tanshinone IIA Sulfonate
TCM: Traditional Chinese Medicine
TF: Tissue Factor
TFPI: Tissue Factor Pathway Inhibitor
TGF-β: Transforming Growth Factor-beta
THD: Tanhuo Decoction
TIMP-1: Tissue Inhibitor of Metalloproteinases-1
TLR4: Toll-like Receptor 4
TNF-α: Tumor Necrosis Factor-alpha
TNFRSF11A: TNF Receptor Superfamily Member 11A
TOS: Total Oxidant Status
TRAIL-R2: TNF-Related Apoptosis-Inducing Ligand Receptor 2
VA: Ventricular Arrhythmias
VEGF: Vascular Endothelial Growth Factor
Wnt: Wingless/Integrated
α-SMA: α-Smooth Muscle Actin
