# Supplementary material for: The role and therapeutic advances of neutrophils in acute myocardial infarction: from traditional chinese medicine modulation to modern therapeutic strategies
Source: Chin Med. 2025 Dec 5;20:213. doi: 10.1186/s13020-025-01261-4 (PMC12679801; doi:10.1186/s13020-025-01261-4)
Supplement: Supplementary file 1 — Supplementary Material 1 [file 13020_2025_1261_MOESM1_ESM.docx]

| **Source (Latin Name)** | **Chemical Class** | **Extract** | **Tested Compound** | **Study Subject** | **Sample Size** | **Intervention Model** | **Control Treatment** | **Experimental Treatment** | **Outcomes** | **Mechanisms** | **Pathway** | **Ref.** |
| --- | --- | --- | --- | --- | --- | --- | --- | --- | --- | --- | --- | --- |
| **Anti-inflammatory** | | | | | | | | | | | | |
| *Astragalus membranaceus (Fisch.) Bunge* | Triterpenoid saponins | AS-IV | AS-IV in 0.5% CMC | Male C57BL/6J mice | - | LAD ligation | 0.5% CMC (20 mL/kg/day, oral) | AS-IV (40 mg/kg/day, oral) | Inflammatory infiltration, myocardial injury, cardiomyocyte hypertrophy, myocardial fibrosis, and cardiac remodeling ↓ | ROS↓, neutrophil expression↓ | ROS/Caspase-1/GSDMD | (59) |
| *Curcuma longa* | Diarylheptanoids | Curcumin | CC nanoparticles (≥94% purity) | Female Wistar rats | 56 | Isoproterenol-induced MI | Saline (s.c.) | CCNP (100/150 mg/kg/day, oral) | Cardiomyocyte necrosis, edema formation, and inflammatory cell infiltration ↓ | Antioxidant response↑ (inhibition of Malondialdehyde [MDA], Total Oxidant Status [TOS], and Nitrogen Oxides [Nox] levels), pro-inflammatory cytokines (e.g., TNF-α, IL-6, IL-1α, IL-1β) serum levels↓, MMPs (MMP-2, MMP-9) expression↓ | NOS/NO | (60) |
| *Poria cocos* | Polysaccharides | PCP | PCP (≥98% purity) | Male SPF SD rats | 80 | LAD ligation/reperfusion | Saline (oral) | PCP (100/200 mg/kg/day, oral) | Focal necrosis in myocardial tissue, cardiomyocyte swelling, unclear boundaries, and neutrophil infiltration ↓ | IL-1β↓, IL-18↓, neutrophil adhesion↓, oxygen free radical production↓ | Rho-ROCK | (61) |
| *Colchicum autumnale L.* | Alkaloids | Colchicine | Colchicine | Male C57BL/6 mice | - | LAD ligation | Cl-amidine (10 mg/kg/day) | Colchicine (0.1 mg/kg/day, i.p.) | Inflammatory response, NETs formation, and cardiac remodeling ↓ | NOX2/ROS↓, Ca²⁺ influx↓, NETs formation↓ | - | (32) |
|  |  |  |  | Human patients | 5,872 | Clinical AMI | Placebo | 0.5–1.0 mg/day | Risk of MACE ↓ with improved cardiovascular prognosis | C-Reactive Protein (CRP)↓, neutrophil levels↓ | - | (62) |
| *Rabdosia rubescens (Hemsl.) H. Hararubescens* | Diterpenoids | Oridonin | Ori | Male C57BL/6 mice | - | LAD ligation | Ori/MCC950/CY-09 | Saline | Neutrophil infiltration, myocardial infarction area, and myocardial fibrosis ↓ | IL-1β↓, IL-18↓, inflammatory infiltration of myocardial neutrophils↓ | - | (63) |
| *Salvia miltiorrhiza Bge.* | Phenanthrenequinones | STS | STS solution | Male C57BL/6J mice | 56 | LAD ligation | Saline (i.p.) | STS (20.8 mg/kg/day, i.p.) | Angiogenesis ↑, cardiac function ↑, myocardial necrosis ↓, inflammatory cell infiltration ↓, scar size ↓ | α-Smooth Muscle Actin (α-SMA)↓, Collagen I & III↓, inflammatory cell infiltration↓, plasma LDH↓, High Mobility Group Box 1 (HMGB1)↓, Interleukin-1β (IL-1β)↓, Tumor Necrosis Factor-α (TNF-α)↓, and protein expression of these cytokines↓ | - | (64) |
|  |  | Tanshinones | Sweated/non-sweated extract | Rats | 96 | ISO-induced AMI | IMST (6.3 mg/kg) | SSPA/NSPA (1/4 g/kg, oral) | Inflammatory cell infiltration ↓, ECG ST-segment elevation ↓, myocardial infarction area ↓ | Aspartate Aminotransferase (AST)↓, Lactate Dehydrogenase (LDH)↓, Creatine Kinase-MB (CK-MB)↓, Superoxide Dismutase (SOD) activity↓ | PPARα/RXRα/NF-κB | (65) |
| *Veratrum grandiflorum* | Stilbenes | RSV | RSV | Male Wistar rats | 60 | ISO-induced AMI | - | RSV(50/100 mg/kg, oral) | Oxidative stress ↓, myocardial necrosis ↓, interstitial edema ↓, neutrophil infiltration ↓ | Cardiac Troponin T (cTnT)↑, LDH↑, AST↑, inflammatory cytokines (TNF-α, IL-1β, NF-κB, etc.) expression↓ | - | (66) |
| **Anti-fibrotic** | | | | | | | | | | | | |
| *Rabdosia rubescens (Hemsl.) H. Hararubescens* | Diterpenoids | Ori | Ori | Male C57BL/6 mice | - | LAD ligation | Ori/MCC950/CY-09 | Saline | Neutrophil infiltration ↓, myocardial infarction area ↓, myocardial fibrosis ↓ | IL-1β↓, IL-18↓, inflammatory infiltration of myocardial neutrophils↓, NOD-like receptor family, pyrin domain containing 3 (NLRP3) activation↓ | - | (63) |
| *Salvia miltiorrhiza Bge.* | Phenanthrenequinones | STS | STS | STEMI patients | 101 | Clinical trial | Saline | STS (80 mg/day×7d) | Progressive left ventricular remodeling ↓, neutrophil degranulation and vesicle rupture ↓ | Neutrophil elastase↓, myeloperoxidase↓, proteinase 3↓, Neutrophil Gelatinase-Associated Lipocalin (NGAL)↓, MMP-8↓, MMP-9↓, neutrophil infiltration and degranulation↓ | - | (67) |
| *Astragalus membranaceus (Fisch.) Bunge* | Triterpenoid saponins | AS-IV | AS-IV in 0.5% CMC | Male C57BL/6J mice | - | LAD ligation | 0.5% CMC (oral) | AS-IV (40 mg/kg, oral) | Myocardial fibrosis ↓, inflammatory infiltration ↓, myocardial injury ↓, cardiomyocyte hypertrophy ↓, cardiac remodeling ↓ | Fibrosis markers (Collagen I, Collagen III, α-SMA, Fibronectin) expression↓ | ROS/Caspase-1/GSDMD | (59) |
| *Epimedium grandiflorum* | Flavonoid glycosides | ICA | ICA solution | Male SD rats | 100 | Coronary ligation | Saline (2 mL/kg) | ICA (3–20 mg/kg) | Cardiac remodeling ↓, cardiomyocyte apoptosis rate ↓ | Tissue Inhibitor of Metalloproteinases-1 (TIMP-1)↑, B-cell lymphoma 2 (Bcl-2)↑, MMP-9↓, Collagen I/III↓, CD147↓, BCL2-Associated X Protein (Bax)↓, caspase-3↓, cleaved caspase-3↓ | CD147/MMP-9 | (68) |
| **Pro-angiogenic** | | | | | | | | | | | | |
| *Salvia miltiorrhiza Bge.* | Phenanthrenequinones | STS | STS solution | Male C57BL/6J mice | 56 | LAD ligation | Saline (i.p.) | STS (20.8 mg/kg/day, i.p.) | Angiogenesis ↑, myocardial necrosis ↓, inflammatory cell infiltration ↓ | CD31+ vessel density↓, Hypoxia-Inducible Factor-1α (HIF-1α) protein level↓, Vascular Endothelial Growth Factor (VEGF) level↓ | - | (64) |
